# Warming indirectly simplifies food webs through effects on apex predators

**DOI:** 10.1038/s41559-023-02216-4

**Published:** 2023-10-05

**Authors:** Eoin J. O’Gorman, Lei Zhao, Rebecca L. Kordas, Steve Dudgeon, Guy Woodward

**Affiliations:** 1 School of Life Sciences, University of Essex, Colchester, UK; 2 Beijing Key Laboratory of Biodiversity and Organic Farming, College of Resources and Environmental Sciences, China Agricultural University, Beijing, China; 3 Georgina Mace Centre for the Living Planet, Department of Life Sciences, Imperial College London, Ascot, Berkshire, UK; 4 Department of Biology, California State University, Northridge, CA USA

**Keywords:** Food webs, Climate-change ecology, Ecological networks, Freshwater ecology, Ecological modelling

## Abstract

Warming alters ecosystems through direct physiological effects on organisms and indirect effects via biotic interactions, but their relative impacts in the wild are unknown due to the difficulty in warming natural environments. Here we bridge this gap by embedding manipulative field experiments within a natural stream temperature gradient to test whether warming and apex fish predators have interactive effects on freshwater ecosystems. Fish exerted cascading effects on algal production and microbial decomposition via both green and brown pathways in the food web, but only under warming. Neither temperature nor the presence of fish altered food web structure alone, but connectance and mean trophic level declined as consumer species were lost when both drivers acted together. A mechanistic model indicates that this temperature-induced trophic cascade is determined primarily by altered interactions, which cautions against extrapolating the impacts of warming from reductionist approaches that do not consider the wider food web.

## Abstract (web summary)

Using a bioenergetic model and manipulative field experiment along a natural stream temperature gradient, the authors identify a temperature-induced trophic cascade where the presence of fish increases algal biomass and reduces decomposition, but only under warming.

## Main

Controlled experiments in artificial conditions have been crucial for identifying first principles and fundamental constraints that have underpinned theory about the ecological effects of warming^1–4^, but they need to be treated with caution when extrapolating to more complex multi-species systems in the wild. For example, single-species laboratory experiments have repeatedly shown a consistent temperature dependence of growth and metabolism, supporting thermodynamic models such as from the Metabolic Theory of Ecology^5,6^. Laboratory experiments with consumer–resource pairs have similarly revealed a systematic thermal scaling of feeding rates^7^, which are central to predictive models of warming impacts on population dynamics^8^. However, both approaches preclude the potential for warming to alter interactions indirectly through the food web, such as via stronger top-down control leading to the cascading effects seen more recently in some outdoor mesocosm experiments^1–3,9–11^. This can alter the relative importance of green (algal) and brown (detrital) pathways of energy flow^12^, which may erode the stability of the food web^13^. Even for mesocosms, however, the synthetic arenas used and the constraints on community assembly processes, combined with the small-scale and short-term duration of warming (relative to the timescale of climate change) limit the validity of extrapolating to natural systems.

One fruitful avenue of research that can tackle these shortcomings involves embedding manipulative experiments within natural long-term and large-scale temperature gradients that span multiple generations across entire food webs^14,15^. Such conditions are rare in the field, but where they exist, such as in geothermal ecosystems, established communities that have been exposed to intergenerational warming gradients can be manipulated in situ. This provides a powerful means by which some of the common confounds and limitations of observational and experimental studies may be disentangled. We employed this approach in geothermal streams in Hengill Valley, Iceland (Supplementary Fig. 1a,b), which contains numerous small streams of different temperature but similar physical and chemical properties^16–18^, providing a tractable model system for combining experimental and observational gradient approaches. Brown trout are the only predatory fish in the system, and they become progressively larger and more abundant in the warmer streams^19^. This seemingly unexpected finding challenged the general prediction that warming favours the small^20^, but was explained by the greater production of the warmer environment, fuelled by increased nutrient supply^18^. Our previous work has also revealed strong thermal dependencies of community biomass, food web structure and ecosystem functioning^16–19,21^, but manipulative experiments are needed as the next logical step to disentangle the relative importance of direct temperature effects on physiology from indirect food-web effects. This is particularly important given the likelihood for warming to reduce predator-free space^22^, increasing the potential for top-down control on lower trophic levels^11^, which could ultimately lead to simpler communities^22,23^.

In this Article, we constructed a set of fenced enclosures to create paired ‘Fish’ and ‘No fish’ treatment reaches in three cold and three warm streams in the catchment (Supplementary Fig. 1c,d). We measured changes in community biomass, decomposition rate and food web properties over the course of a 5 week experiment to test hypotheses about temperature and top-down control (Table 1). Specifically, we hypothesized (H1) that warming would increase community biomass and decomposition rates during the experiment via direct physiological responses (for example, accelerated growth and metabolism), but would simplify the food web by pushing some species beyond their thermal optima. We also hypothesized (H2) that stronger top-down control by the presence of apex fish predators would trigger trophic cascades (that is, reduced invertebrate biomass, but increased algal biomass), suppress decomposition rates due to lower detritivore biomass, and simplify food web structure due to loss of species and trophic links. Finally, we hypothesized (H3) that higher temperatures would accentuate the effects of apex predators by strengthening the cascading effects, weakening decomposition rates and simplifying the food web even further.

**Table 1:** Hypotheses under investigation

	(a)	(b)	(c)	(d)
Hypothesis	Invertebrate biomass	Algal biomass	Decomposition rates	Food web complexity
H1: Higher temperatures will lead to…	↑	↑	↑	↓
H2: Presence of large apex predators will lead to…	↓	↑	↓	↓
H3: Higher temperatures and apex predators will lead to…	↓↓	↑↑	↓↓	↓↓

Here we test hypotheses related to the main and interactive effects of temperature and apex fish predators on community biomass, decomposition rates and food web structure. Note that H3 emerges from the expectation that warming will strengthen apex predator impacts, rather than additive effects of H1 and H2. Decomposition rates may include both microbial and invertebrate-mediated decomposition.

## Results and discussion

**Fig. 1:**
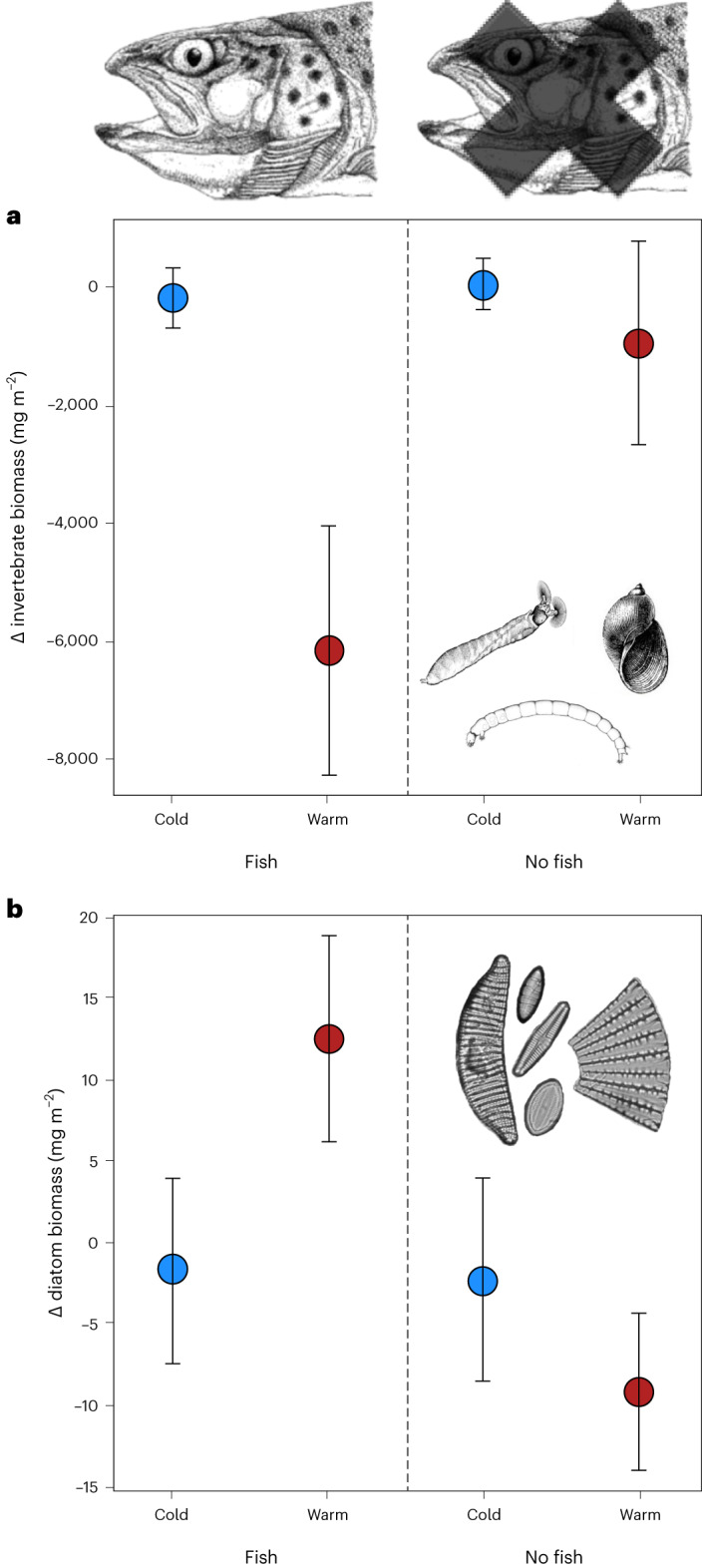
Temperature-induced trophic cascade. **a**,**b**, There was a reduction in the biomass of invertebrates over the course of the experiment (*n* = 60 biologically independent samples) (**a**) and a corresponding increase in the total biomass of diatoms (*n* = 56 biologically independent samples), but only in the presence of fish in the warm streams (**b**). Bars are mean ± standard error; for statistical comparisons between treatments, see Table 2; for a visualization of biomasses in each treatment before and after the experimental manipulation, see Supplementary Fig. 5.

We found no main effects of temperature on invertebrate or algal biomass (in contrast to H1a,b), but invertebrate biomass was strongly suppressed by the fish (supporting H2a), particularly in the warm streams (supporting H3a; Table 2, Fig. 1a and Supplementary Fig. 2). This reduction in biomass was driven largely by suppression of the abundant snail, *Radix balthica* (Supplementary Table 1 and Supplementary Fig. 3a), which is the largest invertebrate in the system and a common prey of brown trout^19,24^. Previous experiments in the Hengill system have shown that *R. balthica* exerts strong top-down control on benthic algae, especially in warmer streams where it dominates the herbivore assemblage^17^. Thus, their suppression here released algae from grazing pressure, increasing diatom biomass and total chlorophyll (supporting H2b), particularly in the ‘Warm–Fish’ treatment (supporting H3b; Table 2, Fig. 1b, Supplementary Fig. 2 and Supplementary Fig. 4). Similar cascading effects have been described for warming experiments in some simple laboratory aquaria and outdoor mesocosms^1,9–11^, but never before for complex systems in the wild. Further taxon-specific findings are described in Supplementary Discussion.

**Table 2:** Statistical output from linear mixed effects models

	Temperature		Fish		Temperature × Fish	
Response variable	*F*	*P*	*F*	*P*	*F*	*P*
Δ invertebrate biomass	0.652	0.465	4.640	0.040	12.06	0.002
Δ diatom biomass	0.444	0.542	3.139	0.092	6.971	0.016
Δ chlorophyll concentration	0.015	0.909	5.088	0.032	7.045	0.013
Microbial decomposition	2.112	0.220	2.189	0.158	6.760	0.019
Invertebrate decomposition	3.013	0.158	1.397	0.255	0.323	0.578
Δ connectance	4.097	0.113	0.536	0.472	4.653	0.043
Δ mean trophic level	0.011	0.920	0.824	0.374	5.603	0.028
Δ consumer–resource ratio	0.398	0.562	0.119	0.734	4.471	0.047

*F* and *P* values are shown for the main effects of temperature category (warm/cold) and fish manipulation (presence/absence) and the interactive effect of these two explanatory variables (Temperature × Fish) on each of the response variables in the experiment.

**Fig. 2:**
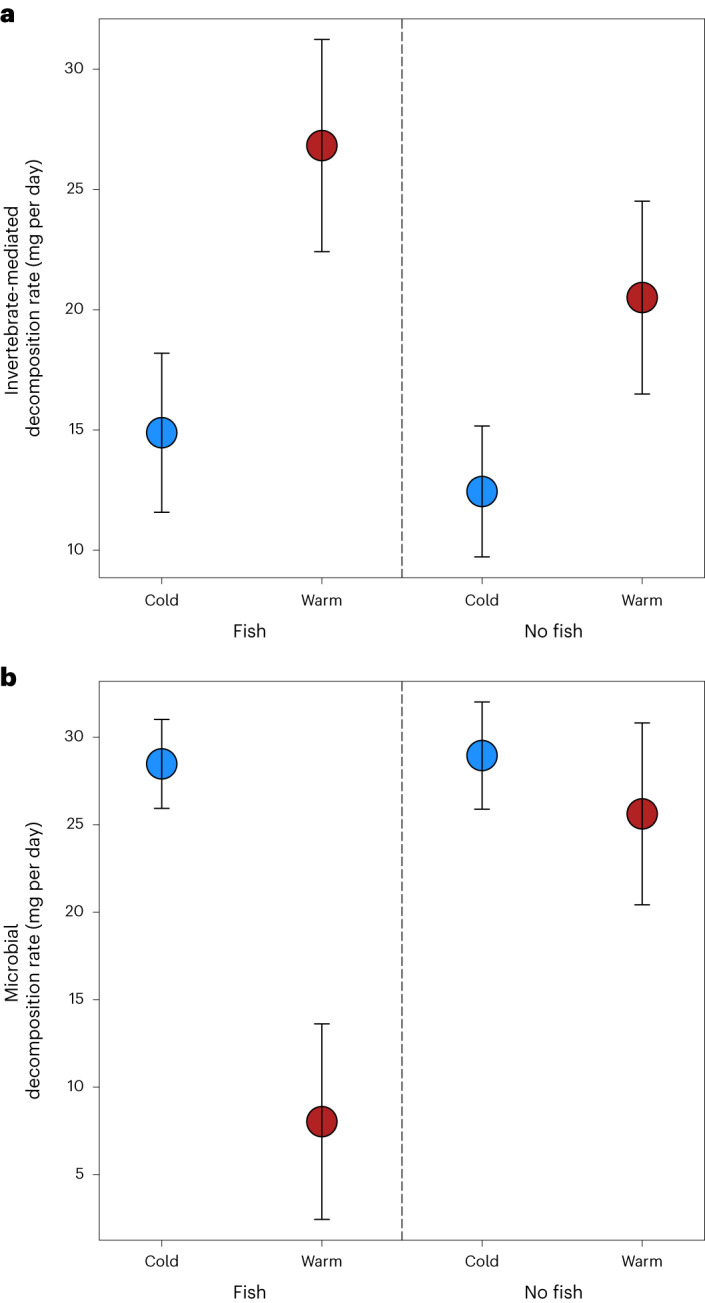
Ecosystem process rates in the experiment. **a**,**b**, There was no effect of temperature or the fish manipulation on invertebrate-mediated decomposition rate (**a**), but microbial decomposition rate was suppressed in the presence of fish in the warm streams (*n* = 36 biologically independent samples in both cases) (**b**). Bars are mean ± standard error; for statistical comparisons between treatments, see Table 2.

Decomposition in freshwater ecosystems is carried out by invertebrate detritivores that directly consume dead organic matter (for example, worms and fly larvae) and microbes that decompose it externally (for example, bacteria and fungi). We found no significant main or interactive effects of temperature and fish presence on invertebrate-mediated decomposition rate (in contrast to H1–3c; Table 2 and Supplementary Fig. 2), even though decomposition rates appeared to be generally higher in the warmer streams (Fig. 2a). This may be driven by the low biomass of invertebrate detritivores due to the absence of leaf litter in the streams, which are instead dominated by algal biofilms and grazing invertebrates^16^—a classic case of the green pathway dominating in stream ecosystems that lack riparian vegetation. Intriguingly, microbial decomposition was slower when fish were present in the warm streams (in support of H3c; Table 2, Fig. 2b and Supplementary Fig. 2), despite these taxa being less directly linked to fish in the food web than the larger detritivores. These decomposers could not have been directly affected by fish or invertebrates because the fine mesh of the litter bags excluded larger consumers. Nutrients stimulate microbial decomposition^25,26^, however, and since our study streams are nitrogen limited^16^, the suppression of invertebrates and their excretion products in the ‘Warm–Fish’ treatment (Fig. 1a) may have been sufficient to reduce microbial activity. This new hypothesis is supported by structural equation modelling (SEM), which highlighted the positive effect of invertebrates on microbial decomposition (Supplementary Fig. 2), and warrants new experiments to test the underlying mechanisms. Note that diatom biomass increased when microbial decomposition decreased, suggesting potential competition between algae and bacteria, but there was no support from SEM for this mechanism (Supplementary Fig. 2).

**Fig. 3:**
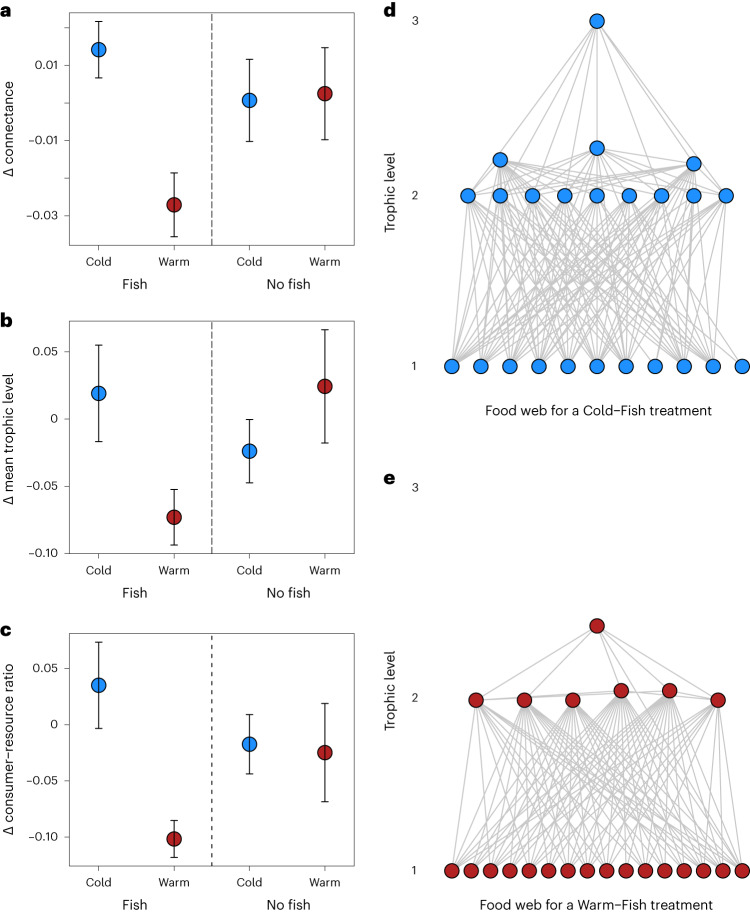
Changes in food web structure during the experiment. **a**–**c**, There was a reduction in connectance (**a**), mean trophic level (**b**) and the ratio of consumer species richness to resource species richness (**c**) during the experiment in the presence of fish in the warm streams (*n* = 52 biologically independent samples in each case). Bars are mean ± standard error; for statistical comparisons between treatments, see Table 2. **d**,**e**, Food webs are visualized for a ‘Cold–Fish’ treatment (**d**) and a ‘Warm–Fish’ treatment (**e**). For a visualization of food web metrics in each treatment before and after the experimental manipulation, see Supplementary Fig. 6 ; for a key to the taxonomic composition of each food web, see Supplementary Fig. 7.

Temperature and apex predators had no main effects on food web structure (in contrast to H1–2d; Table 2), but food webs were less connected and had a lower mean trophic level when fish were present in the warm streams (supporting H3d; Table 2, Fig. 3a and Supplementary Fig. 2). These patterns echo the major changes in biomass and microbial decomposition described above, that is, these different metrics were all modified in the presence of fish in the warm streams. Lower connectance is indicative of a food web that is less resistant to invasion^27^ and less robust to biodiversity loss^28^, while a reduction in mean trophic level is a classic indicator of predator collapse^29^. The latter response is also indicative of a reduction in vertical diversity, that is, loss of trophic levels^30^, which can result in secondary extinctions and reduced ecosystem functioning^31,32^. This provides a potentially novel ecological mechanism to explain the cascading effects in the ‘Warm–Fish’ treatment, whereby reduced omnivorous interactions and intraguild predation could help magnify the top-down control exerted by the apex predator^30^. Indeed, the key driver of the food web changes was a reduced ratio of consumers to resources due to a disproportionate loss of omnivorous invertebrate species in the ‘Warm–Fish’ treatment (Table 2 and Fig. 3c–e). This tendency for trout to feed higher in the food web in warmer streams is reflected by previous stable isotope work in the system showing a higher δ^15^N signature (relative to primary consumers) as stream temperature increases^19^. SEM also revealed the importance of slower microbial decomposition in the presence of fish in the warm streams for decreasing connectance, suggesting that bottom-up (and not just top-down) processes could be simplifying the food web.

**Fig. 4:**
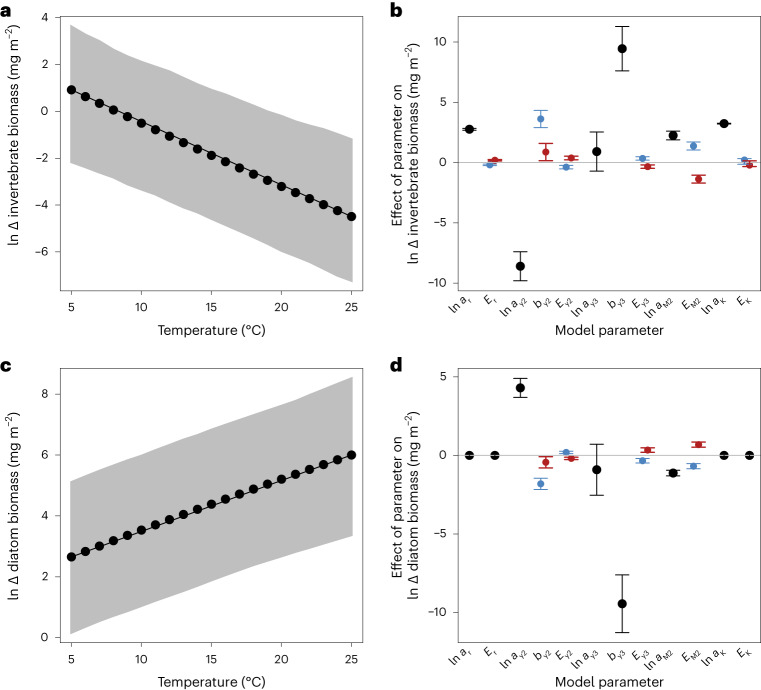
Biomass dynamics from a bioenergetic model. **a**,**c**, There was a reduction in biomass of invertebrates (**a**) and an increase in the biomass of diatoms (**c**) with warming in the presence, compared with the absence, of fish. This result qualitatively reproduces the temperature-induced trophic cascade shown in Fig. 1. Black points are the mean and grey areas indicate the 95% confidence bands from the Monte-Carlo simulations. **b**,**d**, Effects of different model parameters on the change in biomass of invertebrates (**b**) and diatoms (**d**) are also shown, with positive and negative values indicating an increase or decrease in the biomass change relative to the original model, respectively. Parameters shown in black only affect the magnitude of the trophic cascade (that is, the intercept), while parameters shown in blue and red affect its thermal sensitivity (that is, the slope), and thus values are shown for the average temperature of the cold and warm streams, respectively. Parameters associated with the consumption rate of invertebrates (ln *a*_*y*2_) and fish (*b*_*y*3_) had the largest effect on the magnitude of the trophic cascade, while parameters associated with the consumption rate and size of invertebrates (ln *a*_*y*2_ and *E*_*M*2_) had the largest effect on its thermal sensitivity. Bars are mean ± 95% confidence intervals. Note that we log-transformed the change in invertebrate biomass (Δ*B*_2_) as −ln(−Δ*B*_2_) since it is always negative.

To determine the underlying mechanisms driving this temperature-induced trophic cascade, we developed a bioenergetic model describing the dynamics of algal and invertebrate biomass in the presence and absence of fish (Methods). We parameterized the model with data from independent experiments conducted in the system on the growth rate of algae and the consumption rates of invertebrates and fish (Supplementary Methods). The model qualitatively reproduces the decline in invertebrate biomass and increase in algal biomass with warming in the presence of fish (Fig. 4a,c). A sensitivity analysis revealed that parameters associated with consumption rates of the invertebrates and fish determined the strength of the trophic cascade, rather than those associated with direct physiological responses to warming, such as metabolism and growth rate (Fig. 4b,d and Supplementary Figs. 8 and 9). This is one of the first field-model integrations to support the observation that biotic interactions are a stronger mediator of warming impacts on populations than direct physiological changes^33^, though stronger interactions could of course be driven by higher metabolic rates in a warmer environment.

The impacts of warming on natural systems are clearly mediated by the food web and cannot therefore be simply predicted from reductionist approaches that do not take account of indirect effects through multi-species food chains. Multi-trophic micro- and mesocosm experiments still have a crucial role to play in understanding short-term, transient responses of ecological communities to warming, but their findings should be integrated with long-term experiments or space-for-time substitutions that incorporate adaptive responses. Trophic cascades are likely to be especially prevalent under warming in ecosystems dominated by single apex predators^22^, but less so when there is a diverse predator assemblage with greater scope for antagonistic interactions to dampen the consumptive effects on lower trophic levels^31,34^. The simplification of food web structure that we observed due to fish in the warm streams was driven by the loss of larger invertebrates, which tend to be less abundant than smaller organisms and are thus more vulnerable to increased consumption rates^35^. Larger species also tend to have more generalist diets^35^, suggesting that their loss may lead more broadly to shorter food chains and simpler interaction networks.

Local context can play a key role in determining the outcome of warming, with a recent meta-analysis of controlled experiments showing that warming only strengthens top-down control in colder regions, with opposite effects in warmer areas^36^. Our results add weight to this idea and should thus be more relevant for cold, high-latitude ecosystems like our Icelandic study site. In our case, the focal apex predator, brown trout, has scope for increased performance over the temperature range under investigation^19,37^. Warming is more likely to impair the physiology of apex predators closer to their thermal limits, leading to weaker top-down control or even local extinction. Indeed, experiments in tropical tank bromeliads have shown little evidence for interactive effects of warming and apex predators on community structure and ecosystem functioning^38–40^. Instead, there were only main effects of top predator loss on community structure^38,39^ and stability^40^. More multi-trophic warming experiments in other regions are thus needed to determine whether direct physiological effects may override indirect food web effects at lower latitudes^33^.

The absence of any significant main effects of temperature indicates that its direct effects on organismal physiology were insufficient to cause shifts in community biomass, decomposition rates or food web structure over the 5 week duration of the experiment. This may be partly driven by the potential for organisms in the warmer streams to have experienced multi-generational exposure to higher temperatures (that is, the streams have been geothermally heated for many decades, maybe even centuries), and thus exhibit thermal adaptation. Experimental warming or cooling of streams would be needed to overcome this and other limitations of the space-for-time substitution approach. Nevertheless, similar experiments manipulating temperature and apex predators in tropical tank bromeliads showed no direct effects of temperature on community structure^38,39^ or stability^40^, suggesting similar patterns for more transient responses to warming. Notwithstanding the usual caveats that can be applied to field experiments in terms of their scale and complexity (Supplementary Discussion), the lack of direct temperature effects warns against making overly simplistic extrapolations from observational data or reductionist laboratory experiments. These approaches have their merit, but they also need to be placed in the context of the real world if we are to understand and predict future responses to warming. The dearth of field experiments that can plug this gap remains a cause for concern, given how indirect effects through the food web can override temperature responses at the lower organisational levels that we have focused on so far. It is thus critical to consider the trophic structure of natural communities to anticipate future warming impacts, and network-based approaches should be incorporated more broadly into conservation and biomonitoring science to meet that goal^41^.

## Methods

### Experimental design

The study was conducted in the Hengill valley, Iceland (64° 03′ N, 21° 18′ W), which has been intensively studied over the past decade^16–19,21,42,43^. The region consists of numerous spring-fed streams that occur within 1.5 km of each other and have similar physical and chemical properties, yet vary in mean annual temperature from 5 °C to 20 °C due to indirect heating of groundwater through the bedrock^16–18,44^. The system thus acts as a space-for-time substitution to study eco-evolutionary responses of natural ecosystems at the endpoint of warming, but precludes transient responses that could occur during the warming process. Nevertheless, a whole stream warming experiment from the system showed that changes in community composition along the stream temperature gradient are similar to actual warming of a stream^45^.

A field experiment was carried out in six geothermally heated streams in Hengill Valley. A split-plot experimental design was employed, with two levels of temperature (cold and warm) as the main plot crossed with two levels of a fish manipulation (presence and absence of brown trout, *Salmo trutta*) as the subplots within each of the main plots, for a total of four treatments with three replicates of each (Supplementary Fig. 1a). There were three streams in each temperature category (Supplementary Fig. 1b), yielding a mean temperature (± standard deviation, s.d.) over the course of the experiment of 6.8 ± 1.4 °C for the cold streams and 13.5 ± 2.4 °C for the warm streams.

Fish were manipulated by constructing three fences in each stream from metal rebar and extruded plastic netting (10 mm mesh), with each fence separated by a 15 m reach (Supplementary Fig. 1c,d). The average width of the streams in the experiment was 1.5 m, equating to enclosure sizes of approximately 22.5 m^2^. While the mesh size controlled the presence or absence of fish in the experiment, it permitted drifting invertebrates to pass through the fences, which could have led to some upstream (that is, non-treatment) biomass influencing each of the reaches. However, this is a natural process in flowing waters and must be considered a source of unavoidable ‘background noise’ to the experimental treatments.

Electrofishing was carried out on 20 August 2012 to remove any pre-existing brown trout from the experiment, with at least three passes performed on every reach. Ten fish were removed from one of the warm streams (IS6), and six were removed from one of the cold streams (IS14). Fish have also been recorded in IS3 (warm) and IS13 (cold) in past sampling of the system, but never in IS9 (warm) or IS11 (cold)^17–19^. Fish for use in the experiment were electrofished from another stream in the system, IS12, which had an intermediate temperature to the experimental streams (10.5 °C; Fig. 1b). While this precludes adaptive responses of fish to the temperature treatments over the longer term and thus equates more to shorter term, transient responses to temperature, it was a necessary compromise since fish were not already present in every experimental stream in the system. A total of 42 fish with a mean ± s.d. fork length of 17.5 ± 3.3 cm were captured on 22 August and distributed evenly among the ‘Fish’ reaches in the six streams (that is, seven fish per stream). The experimental density of ~0.3 m^−2^ was chosen to match typical densities of brown trout in the catchment^18,19^. Note that the ‘Fish’ reaches were always established downstream of the ‘No fish’ reaches to minimize the chances of fish kairomones eliciting anti-predator behaviour among benthic invertebrates in those treatments^46,47^.

An electrofishing survey was conducted on 7 September after heavy rainfall to ensure that the experimental treatments were still intact. No brown trout were found in any of the ‘No fish’ reaches, although some fish were missing from the ‘Fish’ reaches in each of the other streams. To restore the experimental densities, two new fish from IS12 were added to IS3, 6, 11 and 14, while three fish were added to IS9 and 13. The experiment was terminated after exactly 5 weeks on 26 September. Electrofishing was again performed, with no brown trout found in any of the ‘No fish’ reaches and five, six, three, five, four and six fish recovered from IS3, 6, 9, 11, 13 and 14, respectively. These fish were returned to the stream from which they were originally captured (IS12).

### Sampling

Invertebrates and benthic algae were sampled on 21 August and 25 September, that is, the day before the fish were added to the experiment at the beginning and the day before they were removed from the experiment at the end. Invertebrates were collected by taking five Surber samples (14 × 13.5 cm quadrat; 250 μm mesh) per experimental reach and preserving them in 70% ethanol. Benthic algae were sampled by taking two scrapes of a 2.3 × 3.5 cm micro-quadrat from each of five rocks per reach. We preserved one scrape in stream water with 2% Lugol’s solution for later identification of diatoms. We preserved the second scrape in 96% ethanol, immediately storing it in a black plastic bag, which was placed in a dark fridge at 4 °C upon returning to the lab. Chlorophyll pigments were allowed to extract for an 18 h period before analysis on a DR5000 Hach-Lange spectrophotometer following established methodologies, including a correction for phaeophytin^48^.

We quantified decomposition in the experiment using coarse mesh (5 mm) and fine mesh (250 μm mesh) litter bags. We placed 3.00 g of dried grass (*Carex* spp.) into each bag before sealing them. Note that there are no trees at our study site, so grass represents the major allochthonous input to the streams^16^. Three metal rebars were hammered 20 cm into the sediment in each experimental reach, with one coarse and one fine mesh litter bag attached near the base of each rebar with a cable tie. The litter bags were placed in the streams on 22 August and collected on 26 September. The grass was removed from each litter bag, dried at 80 °C for 48 h, and weighed. Litter breakdown rates (mg per day) were calculated as the initial minus final weight of grass in the litter bags divided by the duration of the experiment (35 days). Microbial decomposition was taken as the breakdown rate in the fine mesh bags, while invertebrate decomposition was the difference between the breakdown rate in each pair of coarse and fine mesh bags.

### Biomass estimation

Invertebrates and diatoms were identified to species level in line with previous studies from the Hengill system^17,18^. The biomass (mg m^−2^) of each species was estimated as mean body mass multiplied by total abundance. The abundance of every invertebrate species was enumerated using a CETI Vari Zoom 10 stereo microscope and scaled by Surber quadrat area (m^−2^). Photographs of every individual identified were taken with a CETI 1.3 megapixel digital USB camera at 80–800× magnification and one linear dimension was measured in Image J^49^ (*n* = 11,053 individuals from 31 species). Published length–weight relationships were used to estimate dry body mass (mg) from the linear measurements^18^.

Diatom frustules were cleared of organic matter with 65% nitric acid, dried, and mounted on slides with naphrax. Abundances were estimated by counting the number of individuals of each species along a 15 × 0.1 mm transect of each slide, ensuring a transect contained at least 150 individuals. The sample dilution, transect area and micro-quadrat area were used to calculate the abundance of each species (m^−2^). Photographs of every individual diatom were taken with the above camera mounted on a CETI Magnum-B phase contrast microscope at 1,000× magnification. Two linear dimensions were measured in Image J^49^ (*n* = 25,248 individuals from 64 species). Every diatom species was assigned a shape, and cell biovolume (μm^3^) was calculated according to associated formulae^50^. Cell carbon content was estimated from published cell volume to cell carbon relationships^51^ and converted to dry mass (mg) assuming an average carbon by dry weight content of 19% per cell^52^.

### Food web construction

We aimed to construct five localized food webs for each experimental reach by pairing the list of invertebrate species from each Surber sample with the list of diatom species from the nearest rock scrape. Food webs were constructed from an established database of 49,324 gut content observations from the Hengill streams, supplemented with literature-based feeding links when yield-effort curves revealed the diet of consumers to be incomplete^21^. Fifty-eight per cent of feeding links were directly observed in each specific stream, 35% of links were inferred from direct observations in other streams in the Hengill system, and just 7% of links were inferred from the literature. A food web link was only included in the current study if both species were found in the paired Surber and rock scrape samples. This procedure ensured that temperature could alter the dietary preferences of the consumers, rather than solely inferring diet from the presence or absence of potential prey. Nevertheless, there was no comparison of invertebrate diets in the presence and absence of brown trout, precluding the possibility that behavioural factors such as fear of predation could have altered their diets. Localized food webs were analysed using the ‘cheddar’ package in R 3.5.0 (ref. ^53^). We computed the following metrics: species richness (*S*), link richness (*L*), linkage density (*L*/*S*), directed connectance (*L*/*S*^2^), mean trophic level (using the ‘ShortWeightedTrophicLevel’ function^53^) and the ratio of consumers to resources (that is, the number of consumer species divided by the number of resource species).

### Statistical analysis

To account for background changes through time in the experiment, we subtracted the mean value of each response variable across the replicates (that is, Surber samples, rock scrapes and litter bags) in an experimental reach at the start of the experiment from the value of that response variable in each replicate in the same experimental reach at the end of the experiment. Thus, if the change in a response variable over the course of the experiment was significantly greater in one treatment compared with another, that difference would be due to the treatment and not natural processes such as growth, migration and death.

We analysed the change in all our response variables over the course of the experiment with linear mixed effects models (‘lme’ function in the ‘nlme’ package of R), where temperature (warm, cold) and fish (presence, absence) were the explanatory variables, and fish treatment within stream identity was a random effect. This random structure accounts for the spatial autocorrelation of the replicates within the ‘Fish’ and ‘No fish’ reaches in each stream. The percentage variation explained by the random effects in each model are given in Supplementary Table 2. To deal with heterogeneity in our model residuals, we compared four different variance structures for each model: (1) setting ‘weights = NULL’ in the ‘lme’ function, that is, assuming homogeneity of variance; (2) setting ‘weights = varIdent(form = ~1 | temperature)’; (3) setting ‘weights = varIdent(form = ~1 | fish)’; and (4) setting ‘weights = varIdent(form = ~1 | temperature * fish)’. The latter three arguments implement different variances per stratum for (2) temperature category, (3) the fish manipulation and (4) each temperature by fish combination^54^. We chose the model with the lowest Akaike Information Criterion to decide the best variance structure for each response variable^54^.

### Bioenergetic model

We constructed a bioenergetic population dynamical model (following ref. ^18^) to simulate the change in biomass of the two main trophic groups in our experiment: diatoms (*B*_1_) and invertebrates (*B*_2_). Fish (*B*_3_) were considered as a third group in the model that consumed invertebrates, but their biomass was maintained as a constant across all streams (*B*_3_ = 28,582 mg m^−2^) to reflect the average biomass of fish added to each stream in the experiment. The changes in biomasses (mg m^−2^) of the two groups through time were modelled as follows:

1 $$\frac{{\mathrm{d}}{B}_{1}}{{\mathrm{d}}t}=r{B}_{1}\Big(1-\frac{{B}_{1}}{K}\Big)-{y}_{2}{B}_{1}{B}_{2}$$

2 $$\frac{{\mathrm{d}}{B}_{2}}{{\mathrm{d}}t}={e}_{2}\,{y}_{2}{B}_{1}{B}_{2}-{x}_{2}{B}_{2}-{y}_{3}{B}_{2}{B}_{3}$$

Here *r* is the maximum mass-specific growth rate of diatoms (per day); *K* is their carrying capacity (mg m^−2^); *x*_2_ is the metabolic rate of invertebrates (per day); *y*_2_ and *y*_3_ represent the consumption rates of invertebrates and fish, respectively (m^−2^ per day); and *e*_2_ = 0.45 is the assimilation efficiency when invertebrates consume diatoms^55^. For modelling the dynamics in the absence of fish, equation (1) remains the same, but equation (2) changes to:

3 $$\frac{{\mathrm{d}}{B}_{2}}{{\mathrm{d}}t}={e}_{2}\,{y}_{2}{B}_{1}{B}_{2}-{x}_{2}{B}_{2}$$

### Equilibrium analysis

We directly calculated the equilibrium biomasses, which were the model-predicted biomasses of the trophic groups at model equilibrium (that is, the steady state of the dynamical system). Considering that the equilibrium biomasses of the two groups were always positive in the experiment, and solving for $\frac{{\mathrm{d}}{B}_{1}}{{\mathrm{d}}t}=\frac{{\mathrm{d}}{B}_{2}}{{\mathrm{d}}t}=0$, we obtain a meaningful equilibrium point for the system with fish:

4 $$\left\{\begin{array}{rcl}{B}_{1}^{\ast } & = & \frac{{x}_{2}+{y}_{3}{B}_{3}}{{e}_{2}\,{y}_{2}}\\ {B}_{2}^{\ast } & = & \frac{r}{{y}_{2}}\Big(1-\frac{{B}_{1}^{\ast }}{K}\Big)=\frac{r}{{y}_{2}}\Big(1-\frac{{x}_{2}+{y}_{3}{B}_{3}}{{e}_{2}\,{y}_{2}K}\Big)\end{array}\right.$$

The equilibrium point without fish is:

5 $$\left\{\begin{array}{rcl}{B}_{1}^{\#} & = & \frac{{x}_{2}}{{e}_{2}\,{y}_{2}}\\ {B}_{2}^{\#} & = & \frac{r}{{y}_{2}}\Big(1-\frac{{B}_{1}^{\#}}{K}\Big)=\frac{r}{{y}_{2}}\Big(1-\frac{{x}_{2}}{{e}_{2}\,{y}_{2}K}\Big)\end{array}\right.$$

We prove both of these equilibrium points are stable in Supplementary Methods, which means the dynamical systems would converge to the equilibrium biomasses we calculated. The difference in biomass between the systems with and without fish is:

6 $$\left\{\begin{array}{c}\Delta {B}_{1}={B}_{1}^{\ast }-{B}_{1}^{\#}=\frac{{y}_{3}{B}_{3}}{{e}_{2}\,{y}_{2}}\\ \Delta {B}_{2}={B}_{2}^{\ast }-{B}_{2}^{\#}=-\frac{r\,{y}_{3}{B}_{3}}{{e}_{2}\,{y}_{2}^{2}K}\end{array}\right.$$

### Model parameterization

We parameterized equation (6) using growth rate data from an algal tile colonization experiment, carrying capacity estimated from a previous study^18^, and consumption rate experiments on invertebrates and fish, all conducted in the Hengill system (for more details, see Supplementary Methods). Note that metabolic rate cancels out when subtracting equation (5) from equation (4) and so does not contribute to the change in biomass of invertebrates or diatoms due to fish. Based on our algal tile colonization experiment, growth rate scales with temperature as follows:

7 $$r={a}_{r}{e}^{\,{E}_{r}(T-{T}_{0})/kT{T}_{0}}$$

where *a*_*r*_ is the growth rate at *T*_0_ (12 °C = 285.15 Kelvin, that is, the average temperature of the experimental streams), *E*_*r*_ is the activation energy describing the Arrhenius increase in growth rate, *k* is the Boltzmann constant (8.618 × 10^−5^ eV per Kelvin) and *T* is stream temperature (for parameter values, see Supplementary Table 3). Based on our previous work^18^, carrying capacity scales with temperature as follows:

8 $$K={a}_{K}{e}^{\,{E}_{K}(T-{T}_{0})/kT{T}_{0}}$$

where *a*_*K*_ is the carrying capacity at *T*_0_ and *E*_*r*_ is the activation energy describing the Arrhenius increase in carrying capacity (for parameter values, see Supplementary Methods). Finally, based on our feeding rate experiments and the Metabolic Theory of Ecology^17,51^, consumption rate scales with body mass and temperature as follows:

9 $${y}_{i}={a}_{yi}{M}_{i}^{\,{b}_{yi}}{e}^{\,{E}_{yi}(T-{T}_{0})/kT{T}_{0}},i=2\,{\rm{or}}\,3$$

where *a*_*yi*_ is the consumption rate at *T*_0_, *b*_*yi*_ is the allometric exponent, *E*_*yi*_ is the activation energy describing the Arrhenius increase in consumption rate, and *M*_*i*_ is the mean body mass of trophic group *i* (for parameter values, see Supplementary Table 4). We considered *M*_3_ = 61,246 mg as the average body mass of fish added to the experimental streams. *M*_2_ increases with temperature in the system^18^, so we estimated the mean body mass of invertebrates at each temperature as:

10 $${M}_{2}={a}_{M2}{e}^{\,{E}_{M\,2}(T-{T}_{0})/kT{T}_{0}}$$

where *a*_*M*2_ is the body mass of invertebrates at *T*_0_ and *E*_*M*2_ is the activation energy describing the Arrhenius increase in body mass (for parameter values, see Supplementary Table 5).

The statistical models performed on the data from each experiment provide a mean and s.d. of the estimate for each parameter. Thus, we ran Monte-Carlo simulations by randomly sampling 1,000 estimates of each parameter from normal distributions with the same mean and s.d. to get the mean and 95% confidence intervals of the equilibrium biomasses for invertebrates and diatoms.

### Sensitivity analysis

We quantified the contribution of each model parameter to the cascading effect of fish on invertebrates and diatoms by individually altering each parameter within the 95% confidence intervals estimated from our experiments. Substituting equations (7)–(10) into equation (6) gives:

11 $$\left\{\begin{array}{rcl}\Delta {B}_{1} & = & \frac{{B}_{3}}{{e}_{2}}\frac{{a}_{y3}{M}_{3}^{{b}_{y3}}}{{a}_{y2}{a}_{M2}^{{b}_{y2}}}{e}^{\Big({E}_{y3}-{E}_{y2}-{b}_{y2}{E}_{M2}\Big)(T-{T}_{0})/kT{T}_{0}}\\ \Delta {B}_{2} & = & -\frac{{B}_{3}}{{e}_{2}}\frac{{A}_{r}{a}_{y3}{M}_{3}^{{b}_{y3}}}{{a}_{K}{a}_{y2}^{2}{a}_{M2}^{2{b}_{y2}}}{e}^{\Big({E}_{r}+{E}_{y3}-2{E}_{y2}-2{b}_{y2}{E}_{M2}-{E}_{K}\Big)(T-{T}_{0})/kT{T}_{0}}\end{array}\right.$$

We defined the cascading effect of fish on diatoms as ln(Δ*B*_1_) and on invertebrates as −ln(−Δ*B*_2_), since Δ*B*_2_ is always negative. The term (*T* − *T*_0_)/*kTT*_0_ exhibits an approximately linear relationship with *T-*12 °C within the experimental temperature range, thus we substituted the term 0.1399 × (*T* − 12) for (*T* − *T*_0_)/*kTT*_0_ in equation (11) to keep the intercept of the model fixed to 12 °C, that is, the average stream temperature in the experiments (Supplementary Fig. 10). The cascading effect of fish would change linearly with temperature according to the ln-transformed version of equation (11). The intercept measures the cascading effect at 12 °C and the slope measures how the cascading effect would change for every 1 °C increase in temperature. Some parameters directly contribute to the magnitude of the cascading effect (that is, altering only the intercept of the equations), while others influence the thermal sensitivity of the cascading effect (that is, altering the slope of the equations).

In equation (11), the directionality of the temperature-induced trophic cascade is governed by two key terms. (1) The effect of warming and presence of fish on invertebrates is governed by *E*_*r*_ + *E*_*y*3_ − 2 × *E*_*y*2_ − 2 × *b*_*y*2_*E*_*M*2_ − *E*_*K*_. If this value is high and positive, the change in invertebrate biomass in the presence compared with the absence of fish declines with warming, whereas if this value is high and negative, the change in invertebrate biomass increases with warming. Increasing *E*_*y*2_, *E*_*M*2_ and *E*_*K*_ or decreasing *E*_*r*_ and *E*_*y*3_ may thus alter the trend from a negative effect of warming on invertebrates in the presence of fish to a positive effect. (2) Similarly, the effect of warming and presence of fish on diatoms is governed by *E*_*y*3_ − *E*_*y*2_ − *b*_*y*2_*E*_*M*2_. If this value is high and positive, the change in diatom biomass in the presence compared with the absence of fish increases with warming, whereas if this value is high and negative, the change in diatom biomass decreases with warming. Increasing *E*_*y*2_ and *E*_*M*2_ or decreasing *E*_*y*3_ may thus alter the trend from a positive effect of warming on invertebrates in the presence of fish to a negative effect.

To assess the effect of each parameter in the model on the cascading effect of the fish, we increased each parameter by 10% in turn and then calculated Δ*B*_1_ and Δ*B*_2_ following equation (11) (denoted as $\Delta {B}_{1}^{{\prime} }$ and $\Delta {B}_{2}^{{\prime} }$, respectively). We quantified the impact of the parameter as the difference in the cascading effect before and after the parameter changed:

12 $$\left\{\begin{array}{c}\mathrm{ln}(\Delta {B}_{1}^{{\prime} })-\,\mathrm{ln}(\Delta {B}_{1})=\,\mathrm{ln}\Big(\frac{\Delta {B}_{1}^{{\prime} }}{\Delta {B}_{1}}\Big)\\ -\,\mathrm{ln}(-\Delta {B}_{2}^{{\prime} })-[-\,\mathrm{ln}(-\Delta {B}_{2})]=\,\mathrm{ln}\Big(\frac{\Delta {B}_{2}^{{\prime} }}{\Delta {B}_{2}}\Big)\end{array}\right.$$

This is the log transformation of the ratio of Δ*B*_1,2_ after increasing the parameter by 10% over Δ*B*_1,2_ before the change. A positive value of the impact indicates the value of Δ*B*_1_ would increase as the parameter increases, or become less negative as the parameter increases in the case of Δ*B*_2_.

### Reporting summary

Further information on research design is available in the Nature Portfolio Reporting Summary linked to this article.

### Supplementary information

Supplementary Information Supplementary methods, results, discussion, Figs. 1–10, Tables 1–5 and references.

Reporting Summary

## Data Availability

The data that support the findings of this study are available from the University of Essex Research Data Repository at 10.5526/ERDR-00000186.

## Code availability

The R code that supports the findings of this study is available from the University of Essex Research Data Repository at 10.5526/ERDR-00000186.
