# Supplementary material for: Warming indirectly simplifies food webs through effects on apex predators
Source: Nat Ecol Evol. 2023 Oct 5;7(12):1983–92. doi: 10.1038/s41559-023-02216-4 (PMC10697836; doi:10.1038/s41559-023-02216-4)
Supplement: Supplementary file 1 — Supplementary methods, results, discussion, Figs. 1–10, Tables 1–5 and references. [file 41559_2023_2216_MOESM1_ESM.pdf]

nature ecology & evolution

Article https://doi.org/10.1038/s41559-023-02216-4

Warming indirectly simplifies food webs through effects on apex predators

In the format provided by the authors and unedited

Table of Contents

2 ………. Supplementary Methods – *Stability analysis*

3 ………. Supplementary Methods – *Algal tile colonisation experiment*

4 ………. Supplementary Methods – *Consumption rate of invertebrates*

5 ………. Supplementary Methods – *Consumption rate of fish*

7 ………. Supplementary Results

7 ………. Supplementary Discussion – *Taxon-specific responses*

8 ………. Supplementary Discussion – *Experimental caveats*

10 ……... Supplementary Figures 1-10

21 ……... Supplementary Tables 1-5

26 ……... Supplementary References

Supplementary Methods

Stability analysis

To determine the stability of the equilibrium biomasses in Equations 4 and 5, we computed the Jacobian matrix of the dynamical model. For the system with fish, the Jacobian matrix is:

$$J = \begin{pmatrix} r - \frac{2r}{K}B_1^* - y_2 B_2^* & -y_2 B_1^* \\ e_2 y_2 B_2^* & e_2 y_2 B_1^* - x_2 - y_3 B_3 \end{pmatrix} \tag{13}$$

Substituting the equilibrium biomasses in Equation 4 into the Jacobian matrix gives the Jacobian determinant:

$$|J| = \begin{vmatrix} -\frac{r}{K}B_1^* & -y_2 B_1^* \\ e_2 r\left(1 - \frac{B_1^*}{K}\right) & 0 \end{vmatrix} \tag{14}$$

The eigenvalues can be computed from solving the following equation:

$$\omega^2 + \frac{r}{K}B_1^* \omega + e_2 y_2 r B_1^* \left(1 - \frac{B_1^*}{K}\right) = 0 \tag{15}$$

We get two eigenvalues when we solve Equation 15:

$$\omega_{1,2} = \frac{-\frac{rB_1^*}{K} \pm \sqrt{(\frac{rB_1^*}{K})^2 - 4e_2 y_2 r B_1^* \left(1 - \frac{B_1^*}{K}\right)}}{2} \tag{16}$$

Since all parameters in Equation 16 are always positive and $0 < B_1^* < K$, we can judge that both ω_1 and ω_2 have negative real parts, and the equilibrium point in Equation 4 is stable.

Similarly, the Jacobian matrix for the system without fish is:

$$J = \begin{pmatrix} r - \frac{2r}{K}B_1^\# - y_2 B_2^\# & -y_2 B_1^\# \\ e_2 y_2 B_2^\# & e_2 y_2 B_1^\# - x_2 \end{pmatrix} \tag{17}$$

Substituting the equilibrium biomasses in Equation 5 into the Jacobian matrix gives the exact same Jacobian determinant as Equation 14, therefore the two eigenvalues would also have negative real parts, and the equilibrium point in Equation 5 is stable.

Algal tile colonisation experiment

To quantify the temperature dependence of benthic algal growth rate, a tile experiment was conducted in four streams in the Hengill system (IS10 = 5.1 °C, IS14 = 9.6 °C, IS9 = 14.0 °C, IS3 = 19.0 °C) over a 43-day period from 12th August to 24th September 2012. A total of 120 ceramic tiles measuring 10 × 10 cm were used in the experiment. A layer of Vaseline (approximately 1 cm in diameter) was placed around the perimeter of each tile to act as a barrier against invertebrate grazers, particularly the snail *Radix balthica*, which is incapable of crawling over petroleum jelly[1,2]. Thirty tiles were spread along the length of each stream, taking care to avoid any macrophytes or rocks covering the upper surface of the tiles. One tile was collected from each stream approximately every three days, resulting in 14 sampling occasions in total. Upon collection, the Vaseline was removed, and the entire surface area of the tile was scrubbed with a toothbrush over a funnel, occasionally rinsing with 96% ethanol. Once the attached biofilm was completely washed into a sample tube, the volume of ethanol was topped up to exactly 50 ml and the tube was placed in a blackened cool box.

Once all four streams were sampled, the tubes were returned to the lab, where they were stored in the dark at 4 °C for approximately 18 hours to allow sufficient time for ethanol extraction of photosynthetic pigments[3]. After this time, the concentration of chlorophyll [mg m^{-2}] in each sample was measured using standard spectrophotometric techniques[3]. We analysed the experimental data with a linear regression obtained from the natural logarithm of Equation 7. Note that the biofilms did not reach carrying capacity during the 43-day duration

of the experiment, so we used the intercept (a_K = 3.233) and temperature-dependence (E_K = -0.304) of carrying capacity that was estimated from a recent modelling study in the Hengill system[4] for parameterising Equation 8 (after changing T_0 in that study from 20 °C to 12 °C).

Consumption rate of invertebrates

To quantify the consumption rate of invertebrates, a series of laboratory experiments were conducted in seven temperature-controlled chambers (4.8–27.5 °C) on 13th, 19th, and 26th June 2017 at the University of Iceland. Biofilms for use in the experiments were cultured on glass coverslips (15 mm diameter) placed in an aquarium containing 2 litres of distilled water, 8 ml of stock medium solution[5], and 250 ml of solution containing diatoms from the genera *Cyclotella*, *Nitzschia*, and *Navicula*, which are all abundant in the Hengill streams[4]. The aquarium was maintained at 21 ± 0.8 °C (mean ± SD) near a north-facing window in a laboratory at the university. 126 coverslips with attached biofilms were removed from the aquarium after each of 25, 30, and 37 days. Each coverslip was placed in the centre of a labelled petri dish containing 25 ml of distilled water for immediate use in the experiments.

The freshwater snail, *Radix balthica*, was chosen as the target invertebrate species for use in the experiment because it is the dominant grazer in the Hengill streams[1,6]. Snails were collected from a cold (IS9), tepid (IS5), and warm (IS8) stream, two days before each experiment started. The snails were placed in aerated aquaria at the university and maintained without food at their home-stream temperature for 48 hours to standardise hunger levels, whilst preventing metabolic down-regulation due to extended starvation[7]. Seven snails were selected from each of five different size classes (estimated by eye from the total range of available snail sizes) from each of the three different home-stream temperatures, equating to 105 snails in total for use in each experiment. One snail from each size class × home-stream temperature combination was then randomly allocated to each of the seven experimental

temperatures and placed on a petri dish containing a coverslip. A photograph was taken of the snails in each petri dish for estimation of body length using ImageJ[8] and subsequent estimation of body mass from an established length-weight relationship for *R. balthica* from the Hengill system[9]. The remaining 21 petri dishes were maintained without snails to act as no-grazing controls, with three of these petri dishes allocated to each of the seven experimental temperatures.

The petri dishes were placed randomly in each of the temperature-controlled chambers, and the three experiments were run for precisely four, two, and three hours, respectively. All snails were immediately removed from the petri dishes to prevent further grazing. The coverslips were subsequently immersed in 10 ml of 96% ethanol and placed in the dark at 4 °C for 18 hours to allow sufficient time for ethanol extraction of photosynthetic pigments[3]. After this time, the concentration of chlorophyll [mg m^{-2}] in each sample was measured using standard spectrophotometric techniques[3]. The mass-specific consumption rate of invertebrates, y_2, was calculated as:

$$y_2 = \frac{C_c - C_e}{tM_2} \quad (19)$$

where C_c is the average concentration of chlorophyll in the three controls for a given chamber, C_e is the concentration of chlorophyll in each experimental unit, t is the duration of the experiment in hours, and M_2 is calculated from Equation 10. We analysed the experimental data with a linear regression obtained from the natural logarithm of Equation 9.

Consumption rate of fish

To quantify the consumption rate of fish, a feeding experiment was conducted in four streams in the Hengill system (IS11 = 4.9-5.9 °C, IS16 = 9.9-10.2 °C, IS1 = 11.5 °C, and IS8 = 17.5-18.9 °C) over a 14-day period from 20th May to 3rd June 2018. At the beginning of the

experiment, 60 brown trout (*Salmo trutta*) were captured by electrofishing three streams (30 from the cold IS12; 30 from the tepid IS1 and IS5). The fish were stored in their natal streams in white plastic buckets (223 × 249 × 315 mm) with two holes measuring approximately 7 cm in diameter covered by 500 µm mesh, allowing in-flow of oxygenated stream water, whilst preventing in- and out-flow of organisms. Buckets were partially submerged in the streams such that at least half the bucket interior was filled with water, leaving the trout access to the surface as in the natural system. Metal rebars were taped to the buckets and hammered into the stream bed to hold the buckets in place, and a rock was placed both inside the bucket and on top of the lid to weigh it down. The fish were supplied with blackfly larvae (Simuliidae) and snails (*Radix balthica*) as food until they were needed in experiments, whereby they were starved for 48 hours to standardise hunger levels, whilst preventing metabolic down-regulation due to extended starvation[7].

Before each experiment, 200 *R. balthica* were hand-collected from an independent stream (IS7), and 20 individuals were added to each of ten empty buckets, which were secured in the experimental stream with metal rebars and rocks. An individual trout was then added to each bucket, ensuring that five buckets contained trout from the cold stream and five contained trout from the tepid streams. The experiments ran for approximately 24 hours, after which time the remaining prey individuals were counted, and fish were released back into their natal streams. The consumption rate of fish, y_3, was calculated as:

$$y_3 = \frac{N_i}{N_f t M_3} \qquad (20)$$

where N_i and N_f are the initial and final density of *R. balthica* in a bucket, t is the duration of the experiment in hours, and M_3 is mean body mass of fish across all streams in the system (61,246 mg). We analysed the experimental data with a linear regression obtained from the natural logarithm of Equation 9.

Supplementary Results

There was a significant decline in algal growth rate as temperature increased in the algal tile colonisation experiment (Supplementary Table 3). There was a significant decrease in the mass-specific consumption rate of the snail, *R. balthica*, as body mass increased and an increase in consumption rate as temperature increased (Supplementary Table 4a). There was also a significant decrease in the mass-specific consumption rate of the brown trout, *S. trutta*, as body mass increased and an increase in consumption rate as temperature increased (Supplementary Table 4b).

Supplementary Discussion

Taxon-specific responses

One of the key findings in the experiment was a decrease in invertebrate biomass and an increase in benthic algal biomass in the presence of fish in the warmer streams (Fig. 1). Whilst we cannot rule out the possibility that invertebrate responses may be due to a combination of consumptive and non-consumptive effects of brown trout (*e.g.* fish kairomones inducing predator avoidance behaviour[10,11]), the absence of clear responses in the "Cold fish" treatment indicates that the effects were largely consumptive (*i.e.* temperature is unlikely to have moderated kairomone effects). In any case, lotic invertebrates typically respond to fish predator cues by decreasing their drift activity[11], thus it is unlikely that reduced invertebrate biomass could have been due to increased downstream migration through the 10-mm mesh of the fences. We also ensured that "No fish" reaches were upstream of the "Fish" reaches to minimise the chances of fish kairomones eliciting anti-predator behaviour amongst benthic invertebrates in "No fish" treatments[10,11].

The temperature-induced trophic cascade was driven largely by a reduction in the biomass of the snail, *Radix balthica*, in the "Warm–Fish" treatment (Supplementary Table 1;

Supplementary Fig. 3a). This snail is the largest invertebrate species in the system[4], a common prey of brown trout[12,13], and has been shown to exert stronger top-down control on benthic algae as temperature increases[1]. The greater biomass of benthic algae in the "Warm–Fish" treatment was driven largely by motile diatoms (Supplementary Table 1; Supplementary Fig. 3d), which are more susceptible to grazing than encrusting forms[14,15] and thus may benefit the most from the suppression of snails. The presence of fish also suppressed the biomass of two blackfly larval species (*Simulium aureum* and *S. vittatum*), but this was independent of temperature (Supplementary Table 1; Supplementary Fig. 3b,c). Blackfly larvae are a preferred prey of brown trout[12,13] and key conduits of energy flux in many running waters, where they can also account for a substantial proportion of the total primary consumer assemblage[16]. Low profile diatoms mirrored the decline of blackfly larvae by increasing in biomass in the presence of fish in both the cold and warm streams (Supplementary Table 1; Supplementary Fig. 3e). Low profile diatoms play a minor role in the diet of blackfly larvae[17,18], thus their success was more likely due to the additional space available on rock surfaces as blackfly larvae numbers decreased. No other invertebrate or diatom taxa exhibited significant responses to the experimental treatments (Supplementary Table 1).

Experimental caveats

The absence of any significant main effects of temperature indicates that its direct effects on organismal physiology were insufficient to predict shifts in community biomass, decomposition rates, or food web structure over the five-week duration of the experiment. It should be noted, however, that while we controlled for the presence or absence of the apex fish predator in our experiment, it would be virtually impossible to exclude all other indirect effects of temperature *via* biotic interactions through the food web. Thus, any expected

effects of higher temperatures on organismal physiology (*e.g.* faster growth increasing biomass, or higher metabolism increasing decomposition rates) may have been offset by stronger competition with, or consumption by, other invertebrate species in the warm streams. The relatively short duration of our experiment may also have contributed to our inability to detect main effects of temperature because it spans less than one generation of most invertebrate species in the system[9]. The labour-intensive maintenance of our experiment in difficult field conditions precluded a longer duration, but this should be considered in future research to determine whether multi-generational (*i.e.* >1 year) responses of populations to higher temperatures accentuate the ecosystem-level consequences.

Supplementary Fig. 1. Design of the field experiment. a, Overview of the experimental design, with six streams divided into two temperature categories (warm and cold) and each stream containing a treatment for the presence or absence of fish. **b,** Map of the experimental streams in the broader Hengill catchment, with stream codes and mean temperature ± standard deviation during the experiment. **c,** Schematic of how the fish manipulation was achieved, with fences dividing the fish and no fish zones into 15 m reaches. **d,** Photograph of a fence used in the experiment.

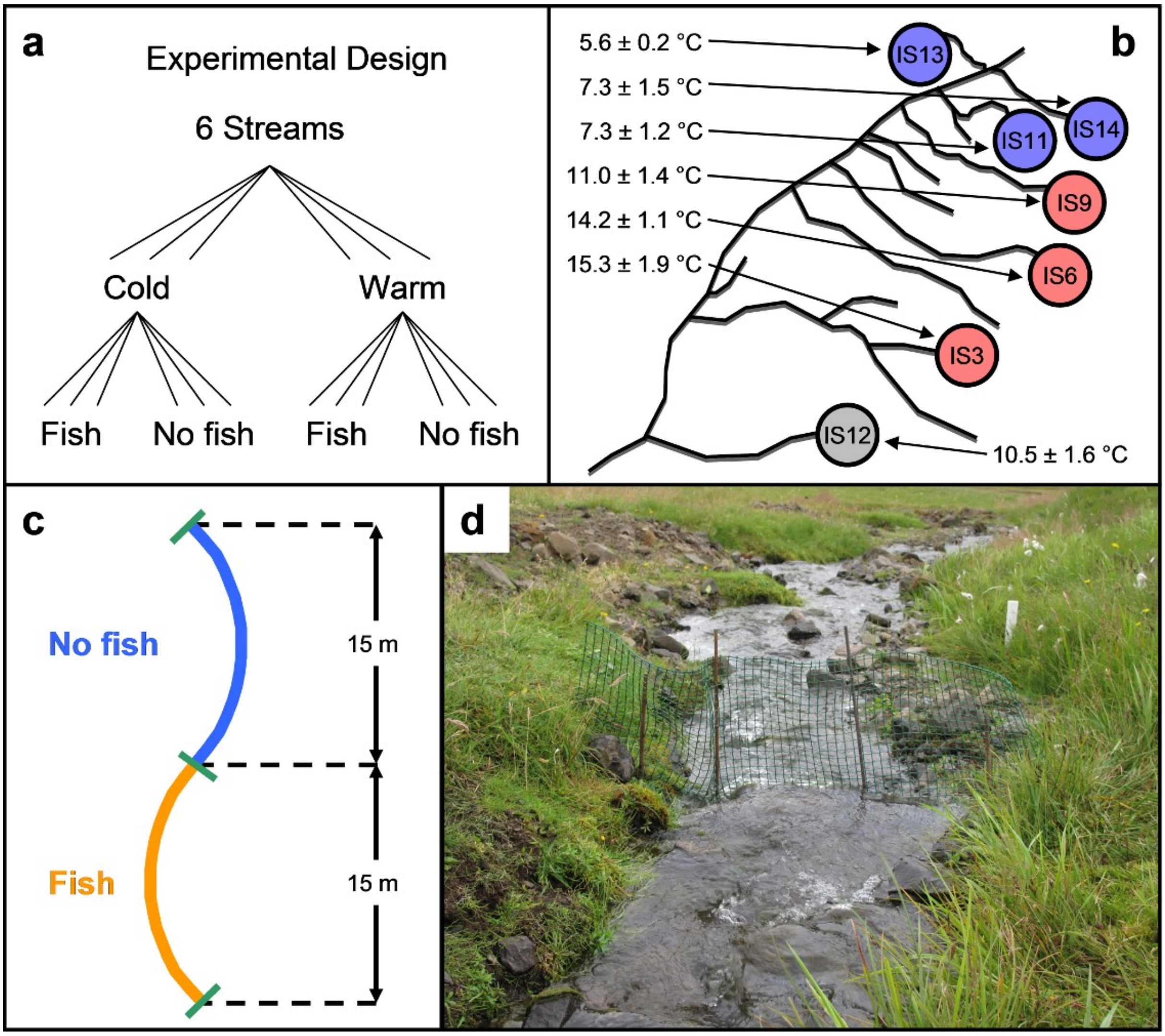

Supplementary Fig. 2. Structural equation model (SEM) for the interactive effects of temperature and the apex fish predator on key responses in the experiment. Piecewise SEM was conducted to test the hypotheses (H1-3) listed in Table 1 and the additional possibility for algal-bacterial competition between diatoms and microbial decomposers, using the same linear mixed effects models described in the main text. Significant positive and negative pathways are represented with black and red lines, respectively, with non-significant pathways in grey. Effect sizes (mean ± SE) relative to the "Cold–No fish" treatment are shown for all significant direct pathways.

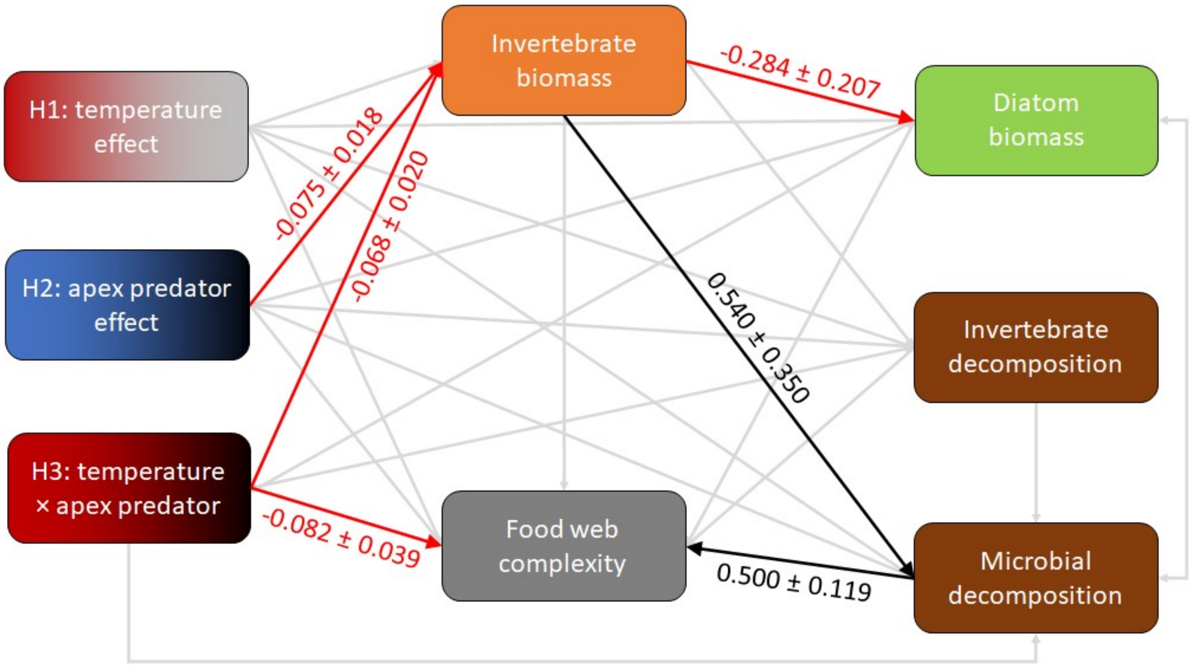

Supplementary Fig. 3. Changes in invertebrate species and diatom group biomass during the experiment. There was a reduction in (**a**) the biomass of the snail *Radix balthica* during the experiment, but only in the presence of fish in the warm streams (*n* = 37 biologically independent samples). There was a reduction in the biomass of the blackfly larvae (**b**) *Simulium aureum* (*n* = 28 biologically independent samples) and (**c**) *S. vittatum* (*n* = 42 biologically independent samples) during the experiment in the presence of fish in both the cold and warm streams. There was an increase in (**d**) the biomass of motile diatoms during the experiment, but only in the presence of fish in the warm streams (*n* = 56 biologically independent samples). There was an increase in (**e**) the biomass of low profile diatoms during the experiment in the presence of fish in both the cold and warm streams (*n* = 56 biologically independent samples). There was (**f**) no difference between any of the treatments in the change in biomass of high profile diatoms during the experiment (*n* = 56 biologically independent samples). Bars are mean ± standard error; see Supplementary Table 1 for statistical comparisons between treatments.

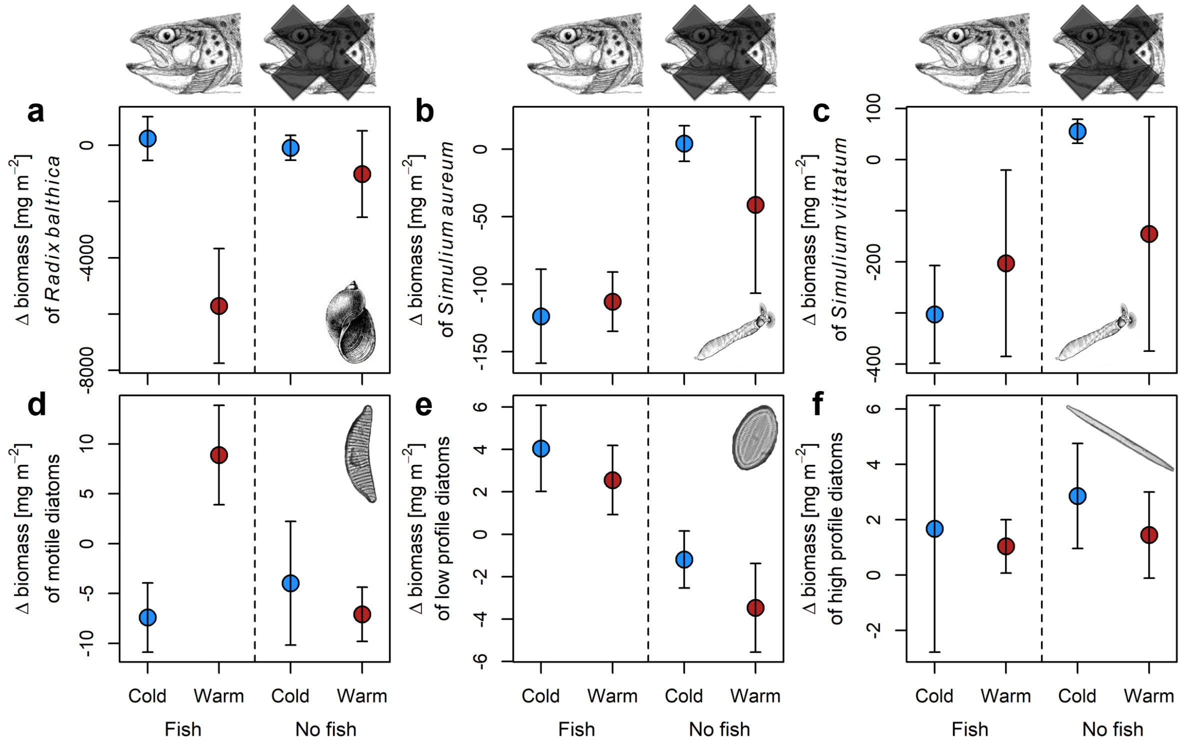

Supplementary Fig. 4. Changes in total chlorophyll during the experiment. There was an increase in the total concentration of benthic algal chlorophyll during the experiment, including diatoms, cyanobacteria, and green algae, but only in the presence of fish in the warm streams (n = 60 biologically independent samples). Bars are mean ± standard error; see Table 1 for statistical comparisons between treatments.

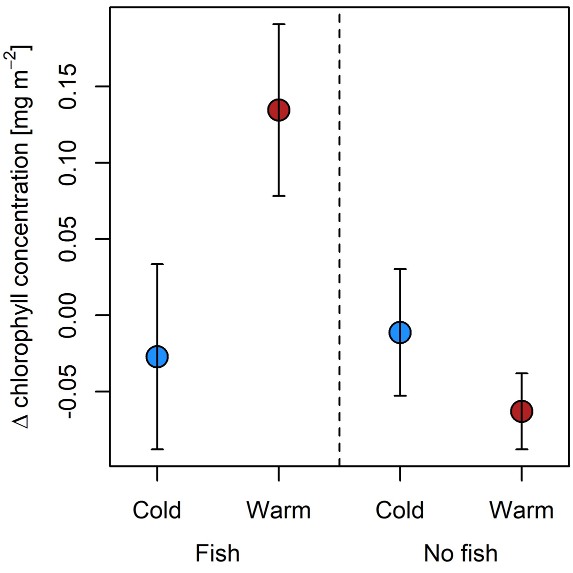

Supplementary Fig. 5. Biomass of invertebrates and diatoms in each treatment before and after the experimental manipulation. (**a**) Invertebrates (*n* = 60 biologically independent samples) and (**b**) diatoms (*n* = 56 biologically independent samples). Bars are mean ± standard error.

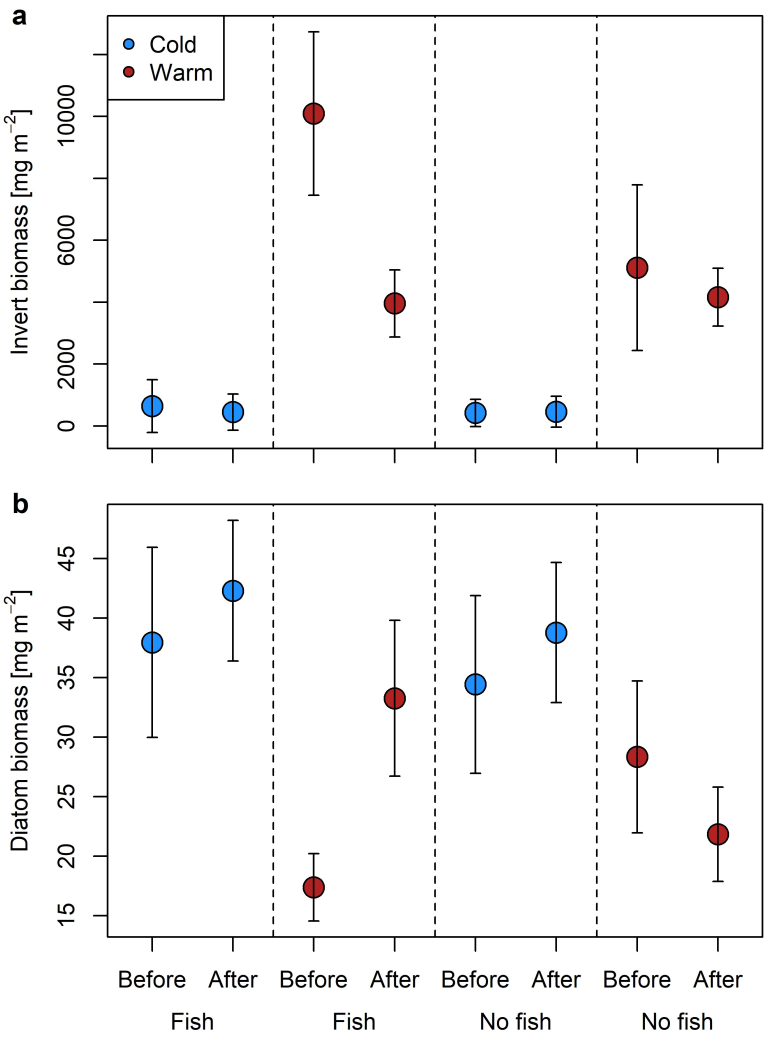

Supplementary Fig. 6. Food web metrics in each treatment before and after the experimental manipulation. (**a**) Connectance, (**b**) mean trophic level, and (**c**) the ratio of consumer species richness to resource species richness (n = 52 biologically independent samples in each case). Bars are mean ± standard error.

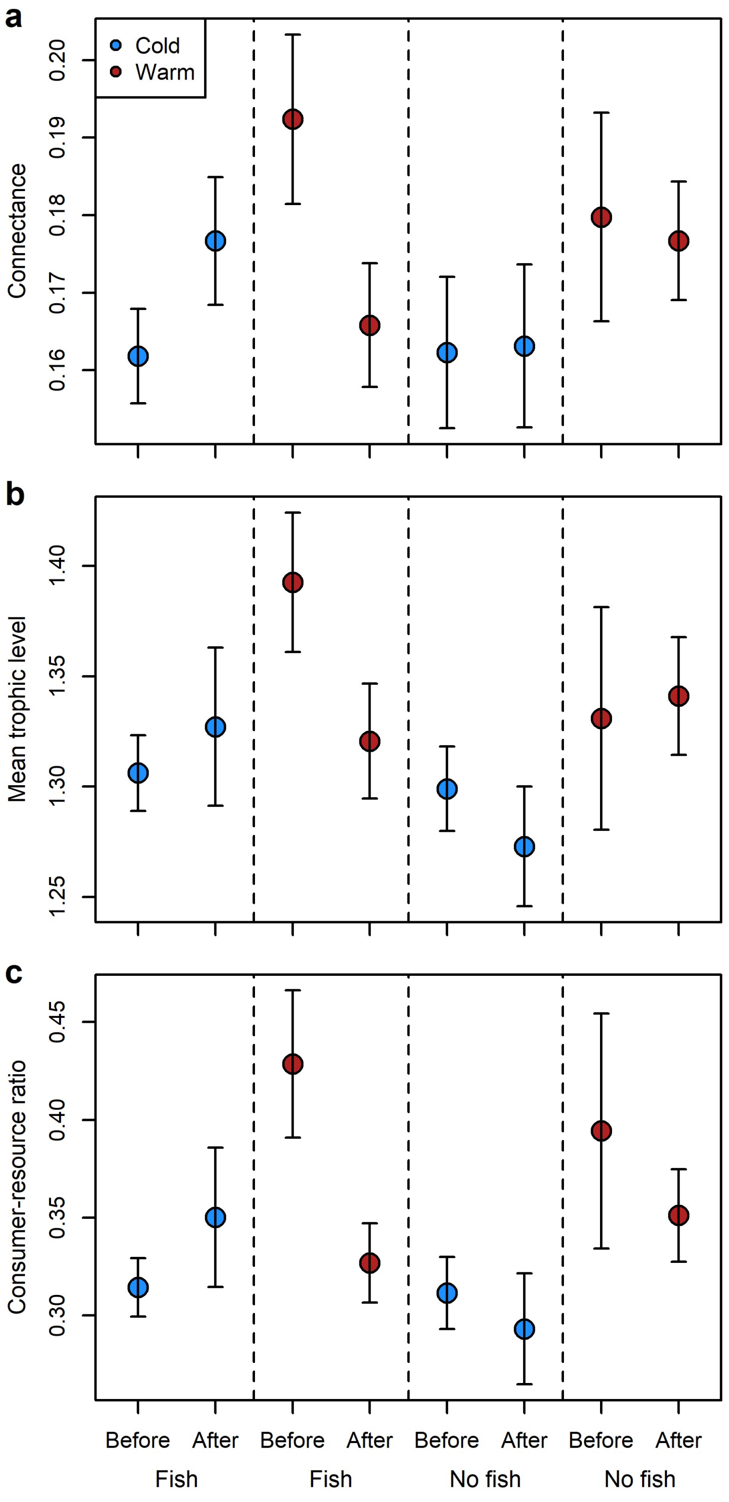

Supplementary Fig. 7. Key to the taxonomic composition of the food webs shown in Fig. 3. Food webs are visualised for (**a**) a "Cold–Fish" treatment and (**b**) a "Warm–Fish" treatment. Key to taxa: 1. *Achnanthes*; 2. *Amphora*; 3. *Chaetocladius* sp.; 4. *Clinocera stagnalis*; 5. *Cocconeis*; 6. CPOM; 7. *Diamesa zernyi*; 8. *Diatoma*; 9. *Dicranota exclusa*; 10. *Epithemia*; 11. *Eukiefferiella claripennis*; 12. *Eukiefferiella minor*; 13. Filamentous algae; 14. FPOM; 15. *Fragilaria*; 16. *Gomphonema*; 17. Green algae; 18. *Limnophora riparia*; 19. *Meridion*; 20. *Metriocnemus* sp. A; 21. *Navicula*; 22. *Nitzschia*; 23. Oligochaeta indet.; 24. *Orthocladius* sp.; 25. *Potamophylax cingulatus*; 26. *Radix balthica*; 27. *Rhoicosphenia*; 28. *Rhopalodia*; 29. *Simulium aureum*; 30. *Simulium vittatum*; 31. *Sperchon glandulosus*; 32. *Synedra*; 33. Terrestrial; 34. *Thienemanniella* sp.

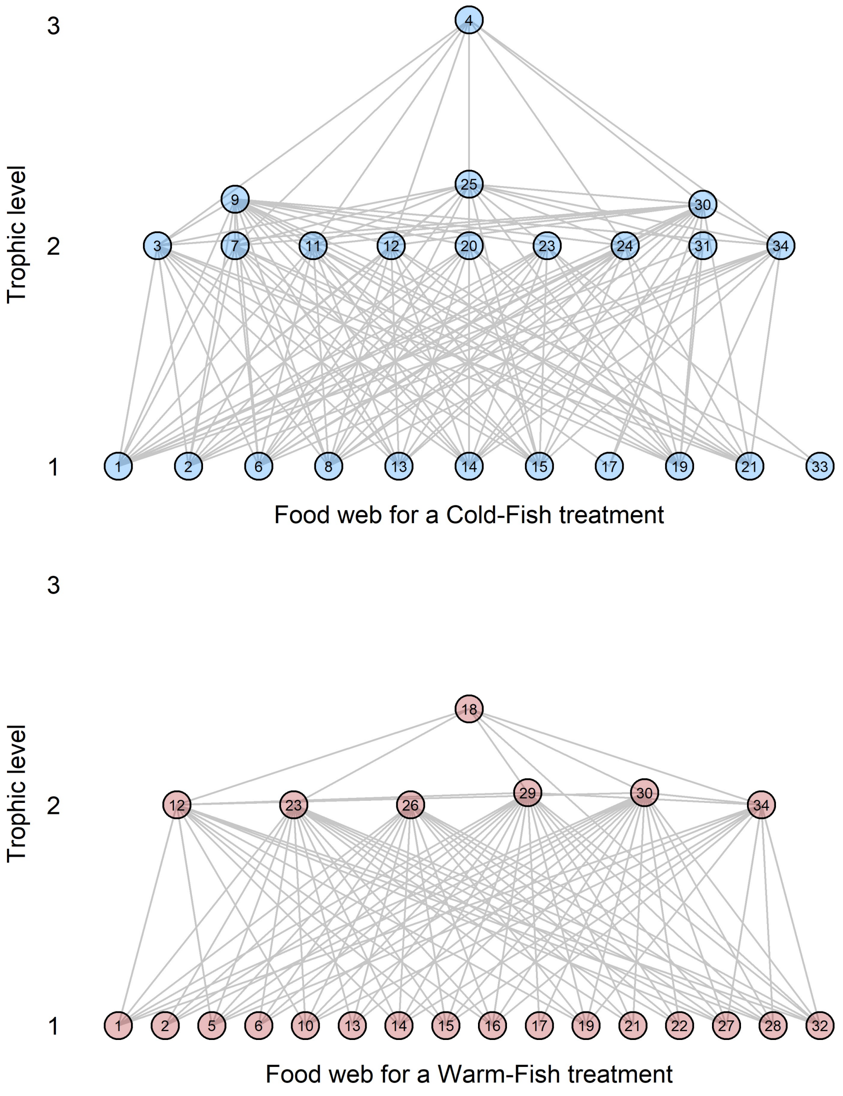

Supplementary Fig. 8. Exploration of the contribution of model parameters to the change in invertebrate biomass across the full temperature gradient in the study system. Black points and lines indicate the effect of temperature on Δ invertebrate biomass for the parameter values used in Equation 11, whilst red and blue lines illustrate how the effect changes for a 10% increase and decrease of each parameter. Exact parameter values for each scenario are provided in the legend within each panel (and in Supplementary Tables 3-5).

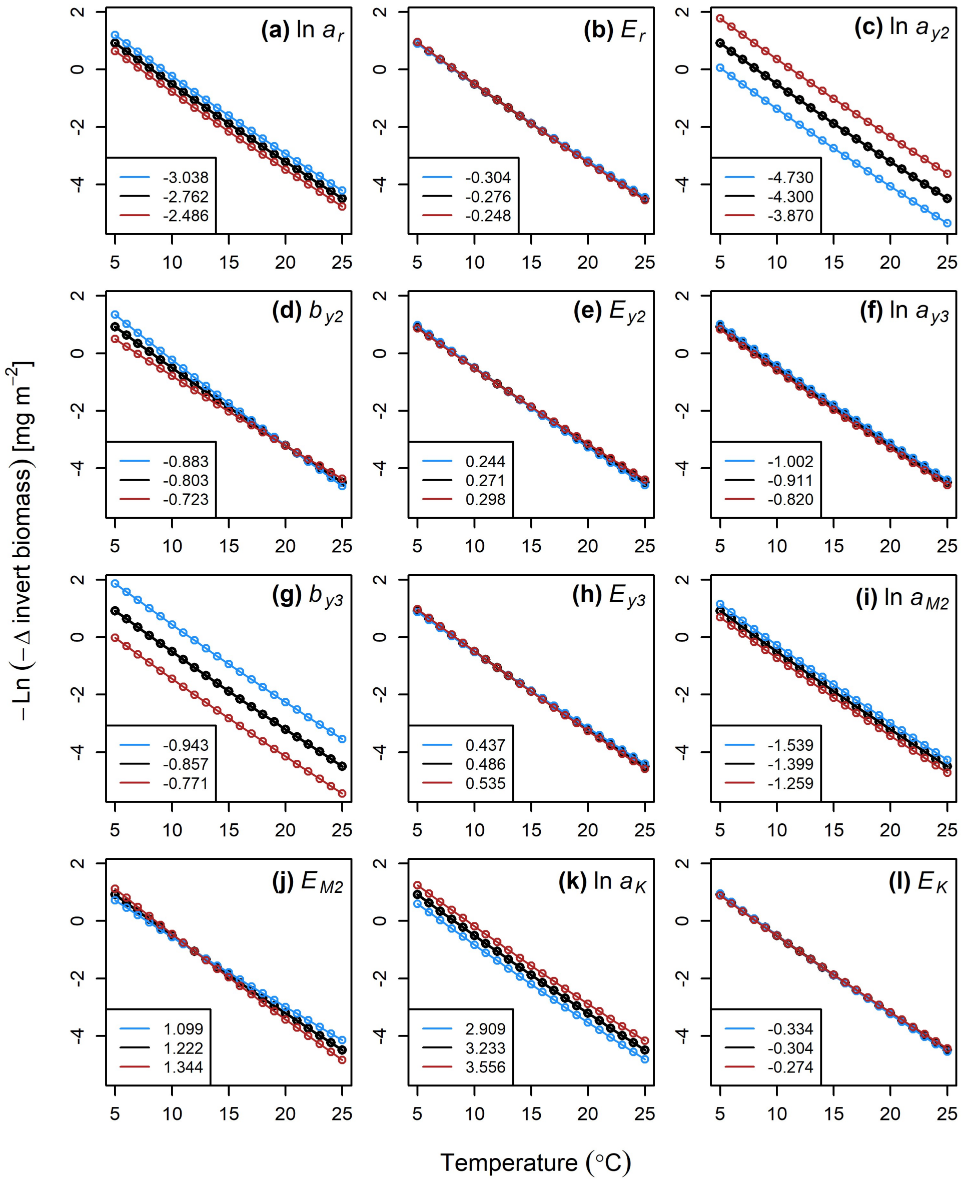

Supplementary Fig. 9. Exploration of the contribution of model parameters to the change in diatom biomass across the full temperature gradient in the study system. Black points and lines indicate the effect of temperature on Δ diatom biomass for the parameter values used in Equation 11, whilst red and blue lines illustrate how the effect changes for a 10% increase and decrease of each parameter. Exact parameter values for each scenario are provided in the legend within each panel (and in Supplementary Tables 3-5).

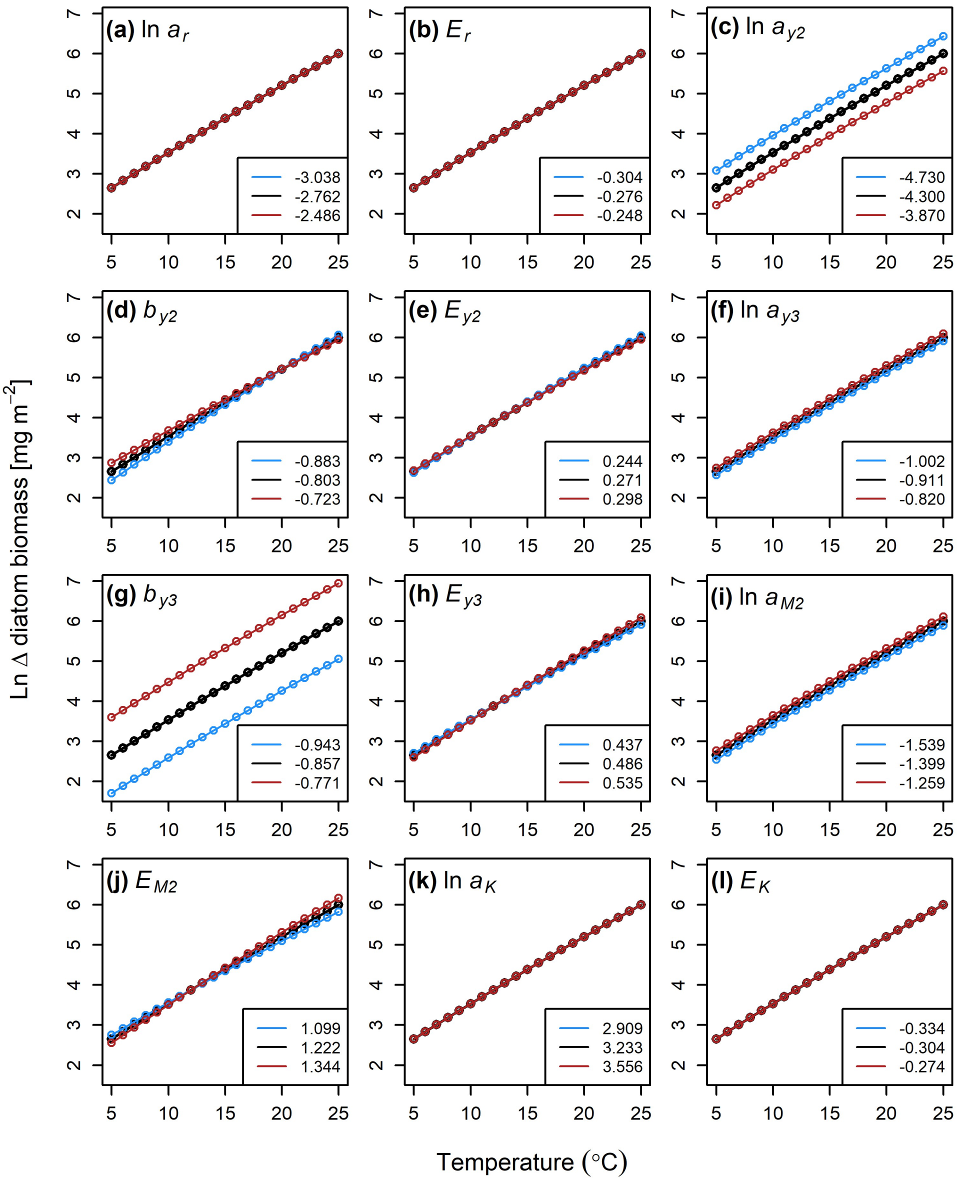

Supplementary Fig. 10. Relationship between the Arrhenius temperature term, (*T*-*T*0)/*kTT*0 [in K], and stream temperature, *T* [in °C]. The equation 0.1399 * (T – 12) is substituted for the Arrhenius temperature term in Equation 11.

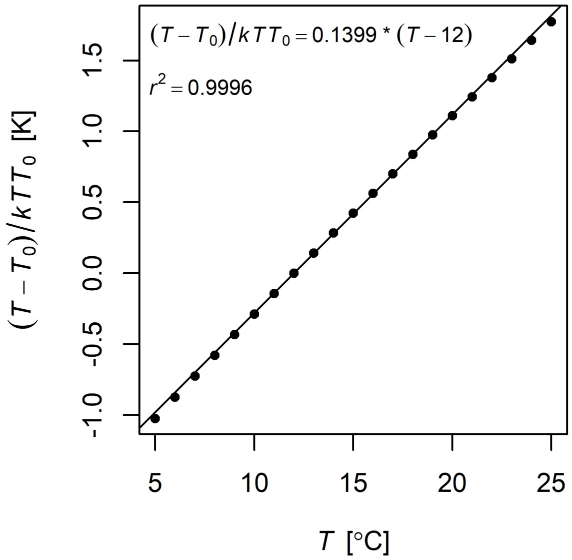

Supplementary Table 1. Statistical output from linear mixed effects models for invertebrate species and diatom groups. *F*- and *p*-values are shown for the main effects of temperature (warm or cold) and fish (presence or absence) and the interactive effect of these two explanatory variables (temp × fish) on the change in biomass of each invertebrate species (with sufficient data) and diatom group in the experiment.

	**temperature**		**fish**		**temp × fish**	
**Response variable**	***F***	***p***	***F***	***p***	***F***	***p***
Δ *Diamesa zernyi* biomass	0.127	0.782	0.781	0.539	0.413	0.636
Δ *Dicranota exclusa* biomass	0.501	0.518	5.003	0.111	1.225	0.297
Δ *Eukiefferiella claripennis* biomass	1.770	0.254	0.006	0.950	0.279	0.691
Δ *Eukiefferiella minor* biomass	1.701	0.262	0.334	0.572	0.270	0.611
Δ *Limnophora riparia* biomass	0.025	0.885	0.881	0.520	0.004	0.961
Δ *Metriocnemus sp.* biomass	<0.001	0.990	0.405	0.570	1.830	0.269
Δ *Micropsectra atrofasciata* biomass	4.631	0.098	0.466	0.525	1.919	0.225
Δ Oligochaeta indet. biomass	0.441	0.543	0.022	0.883	0.123	0.730
Δ *Radix balthica* biomass	0.476	0.540	168.9	<0.001	11.69	0.004
Δ *Rheocricotopus effesus* biomass	1.125	0.349	4.580	0.122	5.129	0.109
Δ *Simulium auereum* biomass	0.052	0.834	9.174	0.023	1.091	0.337
Δ *Simulium vittatum* biomass	0.611	0.478	12.29	0.003	1.474	0.242
Δ *Thienemanniella sp.* biomass	1.288	0.320	1.086	0.310	0.327	0.574
Δ Motile diatoms biomass	0.110	0.757	4.993	0.037	6.480	0.019
Δ Low profile diatoms biomass	0.048	0.837	13.85	0.001	0.410	0.529
Δ High profile diatoms biomass	0.567	0.493	0.160	0.694	0.020	0.889

Supplementary Table 2. Percentage variation explained by the random effects in each model presented in Table 2. Note that the random effects structure consisted of the fish treatment (presence or absence of the apex predator brown trout) nested within streams (a categorical variable with six levels, corresponding to the identity of the six streams in the experiment).

Response variable	stream	fish	residual
δ macroinvertebrate biomass	64	0.052	36
δ diatom biomass	22	35	44
δ chlorophyll concentration	21	23	56
Microbial decomposition	35	0.009	65
Invertebrate decomposition	16	0.016	84
δ connectance	15	0.096	85
δ mean trophic level	45	0.004	55
δ consumer-resource ratio	57	0.002	43

Supplementary Table 3. Statistical output from linear regression model for the temperature dependence of benthic algal growth rate in the Hengill streams. Model estimates and associated standard errors (SE) are shown for the intercept [$\ln(a_r)$] and temperature-dependence [E_r] of the relationship, along with *t*- and *p*-values.

Parameters	Estimate	SE	*t*-value	*p*-value
$\ln(a_r)$	-2.762	0.040	69.40	<0.001
E_r	-0.276	0.043	-6.502	0.023

Supplementary Table 4. Statistical output from linear regression models for the temperature dependence of consumption rate for (a) invertebrates and (b) fish in the Hengill streams. Model estimates and associated standard errors (SE) are shown for the intercepts [ln(a_{y2}) and ln(a_{y3})], size-dependence [b_{y2} and b_{y3}] and temperature-dependence [E_{y2} and E_{y3}] of the relationship, along with *t*- and *p*-values.

Group	Parameters	Estimate	SE	*t*-value	*p*-value
(a) Invertebrates	ln(a_{y2})	-4.300	0.307	-14.02	<0.001
	b_{y2}	-0.803	0.081	-9.901	<0.001
	E_{y2}	0.271	0.054	5.026	<0.001
(b) Fish	ln(a_{y3})	-0.911	0.829	-1.099	0.278
	b_{y3}	-0.857	0.085	-10.12	<0.001
	E_{y3}	0.486	0.103	4.709	<0.001

Supplementary Table 5. Statistical output from linear regression model for the temperature dependence of invertebrate body mass in the Hengill streams. Model estimates and associated standard errors (SE) are shown for the intercept [ln(a_{M2})] and temperature-dependence [E_{M2}] of the relationship, along with *t*- and *p*-values.

Parameters	Estimate	SE	*t*-value	*p*-value
ln(a_{M2})	-1.399	0.115	-12.19	<0.001
E_{M2}	-1.222	0.149	8.189	<0.001

Supplementary References

1 O'Gorman, E. J. *et al.* Impacts of warming on the structure and function of aquatic communities: individual- to ecosystem-level responses. *Advances in Ecological Research* **47**, 81-176 (2012).

2 Hladyz, S., Åbjörnsson, K., Giller, P. S. & Woodward, G. Impacts of an aggressive riparian invader on community structure and ecosystem functioning in stream food webs. *Journal of Applied Ecology* **48**, 443-452 (2011).

3 Steinman, A. D., Lamberti, G. A., Leavitt, P. R. & Uzarski, D. G. in *Methods in Stream Ecology, Volume 1* 223-241 (Elsevier, 2017).

4 O'Gorman, E. J. *et al.* Unexpected changes in community size structure in a natural warming experiment. *Nature Climate Change* **7**, 659-666 (2017).

5 Kilham, S. S., Kreeger, D. A., Lynn, S. G., Goulden, C. E. & Herrera, L. COMBO: a defined freshwater culture medium for algae and zooplankton. *Hydrobiologia* **377**, 147-159 (1998).

6 Friberg, N. *et al.* Relationships between structure and function in streams contrasting in temperature. *Freshwater Biology* **54**, 2051-2068 (2009).

7 Vucic-Pestic, O., Ehnes, R. B., Rall, B. C. & Brose, U. Warming up the system: higher predator feeding rates but lower energetic efficiencies. *Global Change Biology* **17**, 1301-1310 (2011).

8 Abramoff, M. D., Magalhaes, P. J. & Ram, S. J. Image processing with ImageJ. *Biophotonics International* **11**, 36-42 (2004).

9 Hannesdóttir, E. R., Gíslason, G. M., Ólafsson, J. S., Ólafsson, Ó. P. & O'Gorman, E. J. Increased stream productivity with warming supports higher trophic levels. *Advances in Ecological Research* **48** (2013).

10 Pestana, J. L., Loureiro, S., Baird, D. J. & Soares, A. M. Fear and loathing in the benthos: responses of aquatic insect larvae to the pesticide imidacloprid in the presence of chemical signals of predation risk. *Aquatic Toxicology* **93**, 138-149 (2009).

11 Schäffer, M., Winkelmann, C., Hellmann, C. & Benndorf, J. Reduced drift activity of two benthic invertebrate species is mediated by infochemicals of benthic fish. *Aquatic Ecology* **47**, 99-107 (2013).

12 Steingrímsson, S. Ó. & Gíslason, G. M. Body size, diet and growth of landlocked brown trout, Salmo trutta, in the subarctic River Laxá, north-east Iceland. *Environmental Biology of Fishes* **63**, 417-426 (2002).

13 O'Gorman, E. J. *et al.* Temperature effects on fish production across a natural thermal gradient. *Global Change Biology* **22**, 3206-3220 (2016).

14 Connor, M. S., Teal, J. M. & Valiela, I. The effect of feeding by mud snails, Ilyanassa obsoleta (Say), on the structure and metabolism of a laboratory benthic algal community. *Journal of Experimental Marine Biology and Ecology* **65**, 29-45 (1982).

15 Feminella, J. W. & Hawkins, C. P. Interactions between stream herbivores and periphyton: a quantitative analysis of past experiments. *Journal of the North American Benthological Society* **14**, 465-509 (1995).

16 Malmqvist, B., Adler, P. H., Kuusela, K., Merritt, R. W. & Wotton, R. S. Black flies in the boreal biome, key organisms in both terrestrial and aquatic environments: a review. *Ecoscience* **11**, 187-200 (2004).

17 Wallace, J. B. & Merritt, R. W. Filter-feeding ecology of aquatic insects. *Annual review of Entomology* **25**, 103-132 (1980).

18 Gordon, T. A. C., Neto-Cerejeira, J., Furey, P. C. & O'Gorman, E. J. Changes in feeding selectivity of freshwater invertebrates across a natural thermal gradient. *Current Zoology* **64**, 231-242 (2018).
