# Supplementary material for: Warming indirectly simplifies food webs through effects on apex predators
Source: Nat Ecol Evol. 2023 Oct 5;7(12):1983–92. doi: 10.1038/s41559-023-02216-4 (PMC10697836; doi:10.1038/s41559-023-02216-4)
Supplement: Supplementary file 2 — Reporting Summary [file 41559_2023_2216_MOESM2_ESM.pdf]

nature portfolio

Corresponding author(s): Eoin J. O'Gorman

Last updated by author(s): 29/06/2023

Reporting Summary

Nature Portfolio wishes to improve the reproducibility of the work that we publish. This form provides structure for consistency and transparency in reporting. For further information on Nature Portfolio policies, see our Editorial Policies and the Editorial Policy Checklist.

Statistics

For all statistical analyses, confirm that the following items are present in the figure legend, table legend, main text, or Methods section.

n/a	Confirmed	
☐	☒	The exact sample size (*n*) for each experimental group/condition, given as a discrete number and unit of measurement
☐	☒	A statement on whether measurements were taken from distinct samples or whether the same sample was measured repeatedly
☐	☒	The statistical test(s) used AND whether they are one- or two-sided<br>*Only common tests should be described solely by name; describe more complex techniques in the Methods section.*
☐	☒	A description of all covariates tested
☐	☒	A description of any assumptions or corrections, such as tests of normality and adjustment for multiple comparisons
☐	☒	A full description of the statistical parameters including central tendency (e.g. means) or other basic estimates (e.g. regression coefficient) AND variation (e.g. standard deviation) or associated estimates of uncertainty (e.g. confidence intervals)
☐	☒	For null hypothesis testing, the test statistic (e.g. F, t, r) with confidence intervals, effect sizes, degrees of freedom and P value noted<br>*Give P values as exact values whenever suitable.*
☒	☐	For Bayesian analysis, information on the choice of priors and Markov chain Monte Carlo settings
☒	☐	For hierarchical and complex designs, identification of the appropriate level for tests and full reporting of outcomes
☒	☐	Estimates of effect sizes (e.g. Cohen's *d*, Pearson's *r*), indicating how they were calculated

Our web collection on statistics for biologists contains articles on many of the points above.

Software and code

Policy information about availability of computer code

Data collection	No software was used to collect the data.
Data analysis	All statistical analyses were carried out in R 4.0.2.

For manuscripts utilizing custom algorithms or software that are central to the research but not yet described in published literature, software must be made available to editors and reviewers. We strongly encourage code deposition in a community repository (e.g. GitHub). See the Nature Portfolio guidelines for submitting code & software for further information.

Data

Policy information about availability of data

All manuscripts must include a data availability statement. This statement should provide the following information, where applicable:

- Accession codes, unique identifiers, or web links for publicly available datasets
- A description of any restrictions on data availability
- For clinical datasets or third party data, please ensure that the statement adheres to our policy

The data that support the findings of this study will be uploaded to the University of Essex Data Repository upon acceptance of the article.

Research involving human participants, their data, or biological material

Policy information about studies with human participants or human data. See also policy information about sex, gender (identity/presentation), and sexual orientation and race, ethnicity and racism.

Reporting on sex and gender	N/a
Reporting on race, ethnicity, or other socially relevant groupings	N/a
Population characteristics	N/a
Recruitment	N/a
Ethics oversight	N/a

Note that full information on the approval of the study protocol must also be provided in the manuscript.

Field-specific reporting

Please select the one below that is the best fit for your research. If you are not sure, read the appropriate sections before making your selection.

☐ Life sciences ☐ Behavioural & social sciences ☒ Ecological, evolutionary & environmental sciences

For a reference copy of the document with all sections, see nature.com/documents/nr-reporting-summary-flat.pdf

Ecological, evolutionary & environmental sciences study design

All studies must disclose on these points even when the disclosure is negative.

Study description	A field experiment was carried out in six geothermally heated streams in the Hengill valley, Iceland. A split-plot experimental design was employed, with two levels of temperature (cold and warm) as the main plot crossed with two levels of a fish manipulation (presence and absence of brown trout, Salmo trutta) as the subplots within each of the main plots, for a total of 4 treatments with three replicates of each. There were three streams in each temperature category, yielding a mean temperature (± standard deviation, SD) over the course of the experiment of 6.8 ± 1.4 °C for the cold streams and 13.5 ± 2.4 °C for the warm streams. Fish were manipulated by constructing three fences in each stream from metal rebar and extruded plastic netting (10 mm mesh), with each fence separated by a 15 m reach. The average width of the streams in the experiment was 1.5 m, equating to enclosure sizes of approximately 22.5 m^2.
Research sample	Invertebrates and benthic algae were sampled on the day before the fish were added to the experiment at the beginning and the day before they were removed from the experiment at the end (five weeks later). Invertebrates were collected by taking five Surber samples (14 × 13.5 cm quadrat; 250 µm mesh) per experimental reach and preserving them in 70% ethanol. Benthic algae were sampled by taking two scrapes of a 2.3 × 3.5 cm micro-quadrat from each of five rocks per reach. We preserved one scrape in stream water with 2% Lugol's solution for later identification of diatoms. We preserved the second scrape in 96% ethanol, immediately storing it in a black plastic bag, which was placed in a dark fridge at 4 °C upon returning to the lab. Chlorophyll pigments were allowed to extract for an 18-hour period before analysis on a DR5000 Hach-Lange spectrophotometer following established methodologies, including a correction for phaeophytin. We quantified decomposition in the experiment using coarse mesh (5 mm) and fine mesh (250 µm mesh) litter bags. We placed 3.00 g of dried grass (Carex spp.) into each bag before sealing them. Three metal rebars were hammered 20 cm into the sediment in each experimental reach, with one coarse and one fine mesh litter bag attached near the base of each rebar with a cable tie. The litter bags were placed in the streams on 22nd August and collected on 26th September. The grass was removed from each litter bag, dried at 80 °C for 48 hours, and weighed. Litter breakdown rates [mg day^-1] were calculated as the initial minus final weight of grass in the litter bags divided by the duration of the experiment (35 days). Microbial decomposition was taken as the breakdown rate in the fine mesh bags, while invertebrate decomposition was the difference between the breakdown rate in each pair of coarse and fine mesh bags.
Sampling strategy	The number of samples from each treatment reach was based on sample sizes used in previous studies in the Hengill system, which used yield-effort curves to verify the adequacy of sample size, e.g. O'Gorman et al. 2012 Advances in Ecological Research, 47, 81-176.
Data collection	The samples were processed by Eoin O'Gorman and members of his research group, with O'Gorman collating and screening all the datasets into Excel files for subsequent statistical analysis.
Timing and spatial scale	The experiment was conducted from 22nd August to 26th September 2012 in six geothermally heated streams in the Hengill valley, Iceland. There were two treatment reaches in each stream measuring approximately 15 m in length with an average width of 1.5 m, equating to enclosure sizes of approximately 22.5 m^2. Samples were collected at the beginning and end of the experiment to account for background changes through time. Here, we subtracted the mean value of each response variable across the technical replicates (i.e. Surber samples, rock scrapes, litter bags) in an experimental reach at the start of the experiment from the value of that response variable in each technical replicate in the same experimental reach at the end of the experiment. Thus, if the change in a response variable over the course of the experiment was significantly greater in one treatment compared to another, that difference would be due to the treatment and not natural processes such as growth, migration, and death. Fences were cleared of

organic material every 2-3 days and an electrofishing survey was conducted on 7th September after heavy rainfall to ensure that the experimental treatments were still intact, but the experiment was otherwise unperturbed during this time.

Data exclusions

No data were excluded from the analyses.

Reproducibility

The experiment was a huge undertaking in a remote Icelandic valley, so no attempt has been made to repeat it or check the reproducibility of the findings.

Randomization

"Fish" reaches were always established downstream of the "No fish" reaches in each stream to minimise the chances of fish kairomones eliciting anti-predator behaviour amongst benthic invertebrates in those treatments. We accounted for the non-independence of the technical replicates and the spatial autocorrelation of the "Fish" and "No fish" reaches within each stream in our the statistical analyses by using linear mixed effects models ('lme' function in the 'nlme' package of R), where temperature (warm, cold) and fish (presence, absence) were the explanatory variables, and fish treatment within stream identity was a random effect.

Blinding

Samples were processed according to unique codes that were only translated into treatment identifiers once all the data were collected.

Did the study involve field work? ☒ Yes ☐ No

Field work, collection and transport

Field conditions

The study was conducted in the Hengill valley, Iceland, which consists of numerous spring-fed streams that occur within 1.5 km of each other and have similar physical and chemical properties, yet vary in mean annual temperature from 5-20 °C due to indirect heating of groundwater through the bedrock. Air temperatures ranged between 10-20 °C during the experiment, with frequent rainfall and some thunderstorms.

Location

Fieldwork was performed in the Hengill geothermal valley, Iceland (N 64°03; W 21°18) at 350-420 metres above sea level.

Access & import/export

Access to the field site was obtained through collaboration with local researchers from the University of Iceland (Prof Gísli Már Gíslason) and the Marine and Freshwater Research Institute (Dr Jón S Ólafsson). No export permits were required for shipping samples of preserved invertebrates and diatoms from Iceland to the UK, other than filling out customs declaration documentation through the shipping company Eimskip.

Disturbance

The study caused minimal disturbance to the field site. Researchers were careful to stick to established walking trails and to avoid excessive trampling of the vegetation or streams during sampling. The sampling protocols were minimally invasive, with surber samples for invertebrates and rock scrapes for benthic algae. Great care was taken not to cross-contaminate streams in the system.

Reporting for specific materials, systems and methods

We require information from authors about some types of materials, experimental systems and methods used in many studies. Here, indicate whether each material, system or method listed is relevant to your study. If you are not sure if a list item applies to your research, read the appropriate section before selecting a response.

Materials & experimental systems

n/a	Involved in the study	
☒	☐	Antibodies
☒	☐	Eukaryotic cell lines
☒	☐	Palaeontology and archaeology
☐	☒	Animals and other organisms
☒	☐	Clinical data
☒	☐	Dual use research of concern
☒	☐	Plants

Methods

n/a	Involved in the study	
☒	☐	ChIP-seq
☒	☐	Flow cytometry
☒	☐	MRI-based neuroimaging

Animals and other research organisms

Policy information about studies involving animals; ARRIVE guidelines recommended for reporting animal research, and Sex and Gender in Research

Laboratory animals

The study did not involve laboratory animals.

Wild animals

All fish used in the experiment were released back to their natal stream at the end of the experiment. The only animals collected from the field were freshwater macroinvertebrates, which were immediately preserved in 70% ethanol. The sampling protocols were minimally invasive, involving surber samples of a 14 × 13.5 cm quadrat area.

Reporting on sex	Sex of the fish or invertebrates was not considered in the study design because it was not deemed relevant to addressing the hypotheses under investigation.
Field-collected samples	No live organisms were collected at the end of the experiment and so no laboratory housing was needed. Collection and processing of preserved samples is described above in the "Research sample" section.
Ethics oversight	Electrofishing and handling of brown trout in the experiment was performed in collaboration with the Marine and Freshwater Research Institute under their ethical guidelines, permits, and regulations.

Note that full information on the approval of the study protocol must also be provided in the manuscript.
